# A-to-I editing of *Malacoherpesviridae* RNAs supports the antiviral role of ADAR1 in mollusks

**DOI:** 10.1186/s12862-019-1472-6

**Published:** 2019-07-23

**Authors:** Umberto Rosani, Chang-Ming Bai, Lorenzo Maso, Maxwell Shapiro, Miriam Abbadi, Stefania Domeneghetti, Chong-Ming Wang, Laura Cendron, Thomas MacCarthy, Paola Venier

**Affiliations:** 1 Department of Biology, University of Padova, 32121 Padova, Italy; 2 Helmholtz Centre for Polar and Marine Research, Alfred Wegener Institute (AWI), Wadden Sea Station, 25992 List auf Sylt, Germany; 3 Chinese Academy of Fishery Sciences, Yellow Sea Fisheries Research Institute, Qingdao, China; 4 Department of Applied Mathematics and Statistics, Stony Brook University, Stony Brook, NY USA; 5 Istituto Zooprofilattico Sperimentale delle Venezie, 35020 Legnaro, Italy

**Keywords:** ADAR, Malacoherpesvirus, OsHV-1, AbHV-1, Mollusks, Oysters, Abalones, RNA editing, Antiviral responses, A-to-I editing

## Abstract

**Background:**

Adenosine deaminase enzymes of the ADAR family are conserved in metazoans. They convert adenine into inosine in dsRNAs and thus alter both structural properties and the coding potential of their substrates. Acting on exogenous dsRNAs, ADAR1 exerts a pro- or anti-viral role in vertebrates and *Drosophila*.

**Results:**

We traced 4 ADAR homologs in 14 lophotrochozoan genomes and we classified them into ADAD, ADAR1 or ADAR2, based on phylogenetic and structural analyses of the enzymatic domain. Using RNA-seq and quantitative real time PCR we demonstrated the upregulation of one ADAR1 homolog in the bivalve *Crassostrea gigas* and in the gastropod *Haliotis diversicolor supertexta* during *Ostreid herpesvirus-1* or *Haliotid herpesvirus-1* infection. Accordingly, we demonstrated an extensive ADAR-mediated editing of viral RNAs. Single nucleotide variation (SNV) profiles obtained by pairing RNA- and DNA-seq data from the viral infected individuals resulted to be mostly compatible with ADAR-mediated A-to-I editing (up to 97%). SNVs occurred at low frequency in genomic hotspots, denoted by the overlapping of viral genes encoded on opposite DNA strands. The SNV sites and their upstream neighbor nucleotide indicated the targeting of selected adenosines. The analysis of viral sequences suggested that, under the pressure of the ADAR editing, the two *Malacoherpesviridae* genomes have evolved to reduce the number of deamination targets.

**Conclusions:**

We report, for the first time, evidence of an extensive editing of *Malacoherpesviridae* RNAs attributable to host ADAR1 enzymes. The analysis of base neighbor preferences, structural features and expression profiles of molluscan ADAR1 supports the conservation of the enzyme function among metazoans and further suggested that ADAR1 exerts an antiviral role in mollusks.

**Electronic supplementary material:**

The online version of this article (10.1186/s12862-019-1472-6) contains supplementary material, which is available to authorized users.

## Background

Since the early life, cells have been parasitized by self-replicating elements such as viruses [1]. As a result, all cellular organisms have developed antiviral defense mechanisms [2] and the arms race between viruses and their hosts has contributed to shape both their genomes over millions of years [3]. Virus abundances are especially noticeable in marine coastal ecosystems [4] and viruses of different origin are often found in filter-feeding invertebrates such as bivalve mollusks [5–9]. Among the variety of potential pathogens, dsDNA viruses of the *Malacoherpesviridae* family represent a major issue for a number of bivalve and gastropod species, as they have greatly challenged the abalone and oyster aquaculture in the last decades [10–13]. Although the evolutionary history of *Malacoherpesviridae* is largely unknown and this virus family is only distantly related to vertebrate herpesviruses [14–16], the identification of *Malacoherpesviridae-like* sequences in the genome of *Crassostrea gigas* (bivalve), *Capitella teleta* (nematode) and *Branchiostoma spp*. (chordate) suggests a past history of intricate relationships and a possible long lasting co-evolution between these viruses and their hosts [17]. Nowadays, high-throughput sequencing (HTS) supports the investigation of both transcriptional and genomic landscapes, successfully disclosing such features in non-model organisms also during in-vivo infections and providing an unprecedented resolution of molecular host-pathogen interactions [18]. In particular, dual RNA-seq and other HTS approaches have been used to investigate host’s response and the genetic basis for host’s resistance or susceptibility to *Malacoherpesviridae* [17, 19–24]. Although hemocytes have been recently used as an in-vitro model for studying *Malacoherpesviridae* infections [25, 26], the propagation of these viruses is still relying almost exclusively on in-vivo experiments.

The response of multicellular hosts to viral infection is supposed to originate from an ancestral defense system used to control selfish genetic elements [2]. Innate and adaptive defense mechanisms have evolved to prevent the viral entry, to inhibit virus replication and to destroy viral-derived products [27]. In vertebrates, once activated by viral nucleic acids, intracellular receptors lead to the expression and extracellular release of signaling proteins, such as cytokines and interferons (IFNs) and, in turn, IFNs induce the expression of hundreds of interferon-stimulated genes (ISG) altogether shaping a powerful antiviral front line [28]. In the absence of a canonical IFN-mediated response pathway and cell-mediated adaptive immunity, invertebrates rely on inborn defenses. Among them, the recognition and processing of non-self RNAs by RNA interference (RNAi) represents a major defense against viral infections, lately reported in arthropods [29] and suggested to be active also in a gastropod mollusk [30]. How much sequence-specific long dsRNAs can interfere with the viral replication and define an enduring anti-viral state remains to be established in-vivo [31]. The antiviral responses of bivalve mollusks are mediated by cytosolic receptors such as *retinoic acid inducible gene I-like* (RIG-I), *stimulator of interferon gene* (STING) transmembrane proteins, TLRs and a plethora of viral-induced proteins, whose functional roles have been only partially described [32–34]. Among other processes, apoptosis and autophagy likely protect oysters from viral infections [35, 36]. The expression of genes outlining an *interferon-like* pathway was demonstrated in *C. gigas* injected [20] or naturally infected [17] with *Ostreid herpesvirus-1* (OsHV-1). Although an IFN homolog has not yet been identified in bivalves, several ISG homologs such as *viperin*, *2′-5′-oligoadenylate synthase* and *double-stranded RNA-specific adenosine deaminase* (ADAR) have been reported as upregulated upon viral infection [33].

Viral genomes rapidly diversify and evolve owing to a low replication fidelity, as referred for RNA viruses [37, 38], while the replication of DNA viruses is generally more accurate [39] and the evolvability of these viruses probably depend also on other mechanisms [40, 41]. Notably, an increased genetic variation can allow viruses to escape the host defenses [42, 43], but the accumulation of dysfunctional mutations in viral nucleic acids can be exploited as antiviral defense [44, 45]. Editing enzymes such as *tRNA adenosine deaminases* (ADAT), proteins of the *activation induced cytidine deaminase* (AID)/ *apolipoprotein B editing complex* (APOBEC) family and ADARs have been involved in the inactivation of RNA viruses and in the control of retroviruses or retrotransposons, among other processes such as carcinogenesis, diversification of antibodies and editing of various types of RNAs in mammals [46–48]. Proteins of the ADAR family promote the Adenosine to Inosine (A-to-I) conversion by deamination in dsRNAs, resulting in A-to-G (guanine) substitutions which destabilize the dsRNA structure and introduce non-synonymous substitutions [49]. The ADAR gene family likely originated from the ancestral ADAT gene, which seems to be present in all eukaryotes. Differently from ADAT, ADAR homologs have been traced only in metazoans [50, 51], with at least one *adenosine deaminase domain-containing protein* (ADAD1) and three ADARs (ADAR1–3) present in the human genome [52]. The alternative splicing of human ADAR1 results in a long (ADAR1-L, 150 kDa) and in a short form (ADAR-S, 110 kDA), with different cellular distribution: ADAR1-L has been demonstrated to move between cytoplasm and nucleus, whereas ADAR1-S (like ADAR2) has been located in the nucleus [53]. The human genome includes numerous A-to-I editing sites, mostly located in non-coding regions, such as introns and 3′-UTRs, often enriched in *Alu* repeats, as well as in miRNA precursor regions [54]. Among invertebrates, adenosine deaminase activity was reported in Arthropoda (*Drosophila melanogaster*), Crustacea (*Artemia parthenogenetica*), Nematoda (*Caenorhabditis elegans*) and Cephalopoda (*Octopus bimaculoides*) as well as in the earliest-diverging phyla of Metazoa [55–60].

Although ADAR targets adenine, the 5′-flanking nucleotide plays an important role, with the motifs CA, AA and TA being considered strong ADAR-targets whereas the GA dinucleotide has been reported as a weak target in *D. melanogaster* [61]. ADAR-mediated deamination of viral RNAs has been reported for several viruses, like negative-sense RNA viruses, ambisense RNA viruses and DNA viruses [44, 62]. A-to-I substitutions in viral RNAs can be promiscuous or site-selective, depending of the host-virus association [53]. This RNA editing, termed hyper-editing in the case of multiple sequence changes, positively or negatively impacts the virus and influences virus-host interactions [63]. Conversely, the prolonged activity of ADAR on pathogenic viruses can result in the modification in their genomes, to minimize the antiviral host editing activity. Accordingly, the amounts of weak dinucleotide motifs in the genomes of *Zika virus*, *Drosophila Sigma virus* and *Circulating type 1 vaccine-derived poliovirus* have been linked to the evolutionary pressure of RNA editing enzymes [61, 64, 65] whereas an antiviral ADAR-mediated editing has been reported only in the fruit fly against the *Sigma virus* [66] and in shrimp against the *White spot syndrome virus* (WSSV) [67].

Aiming to examine the functional role of ADAR, we searched for ADAR-compatible editing events, namely ADAR editing footprints, in RNA-seq data obtained from oysters and abalones infected in vivo by Malacoherpesviruses. As a result, we detected ADAR homologs in lophotrochozoan genomes and reported them according to phylogenetic and structural analyses. To ascertain whether ADAR1 was modulated during viral infection, we applied RNA-seq and real time quantitative transcription PCR (qRT-PCR) on viral-infected *Crassostrea gigas* and *Haliotis diversicolor supertexta* samples. Exploiting paired DNA and RNA HTS data from the same infected mollusk species, we could map the incidence and the preferential positions of ADAR-editing events in the genomes of the only two *Malacoherpesviridae* viruses known so far (OsHV-1, and Haliotid herpesvirus-1 (AbHV-1). Finally, we comparatively analyzed the whole genomes of these viruses in the hypothesis that these genomes have evolved to minimize host’s ADAR editing.

## Results

### Presence and typical features of *ADAR-like* genes in lophotrochozoans

**Fig. 1:**
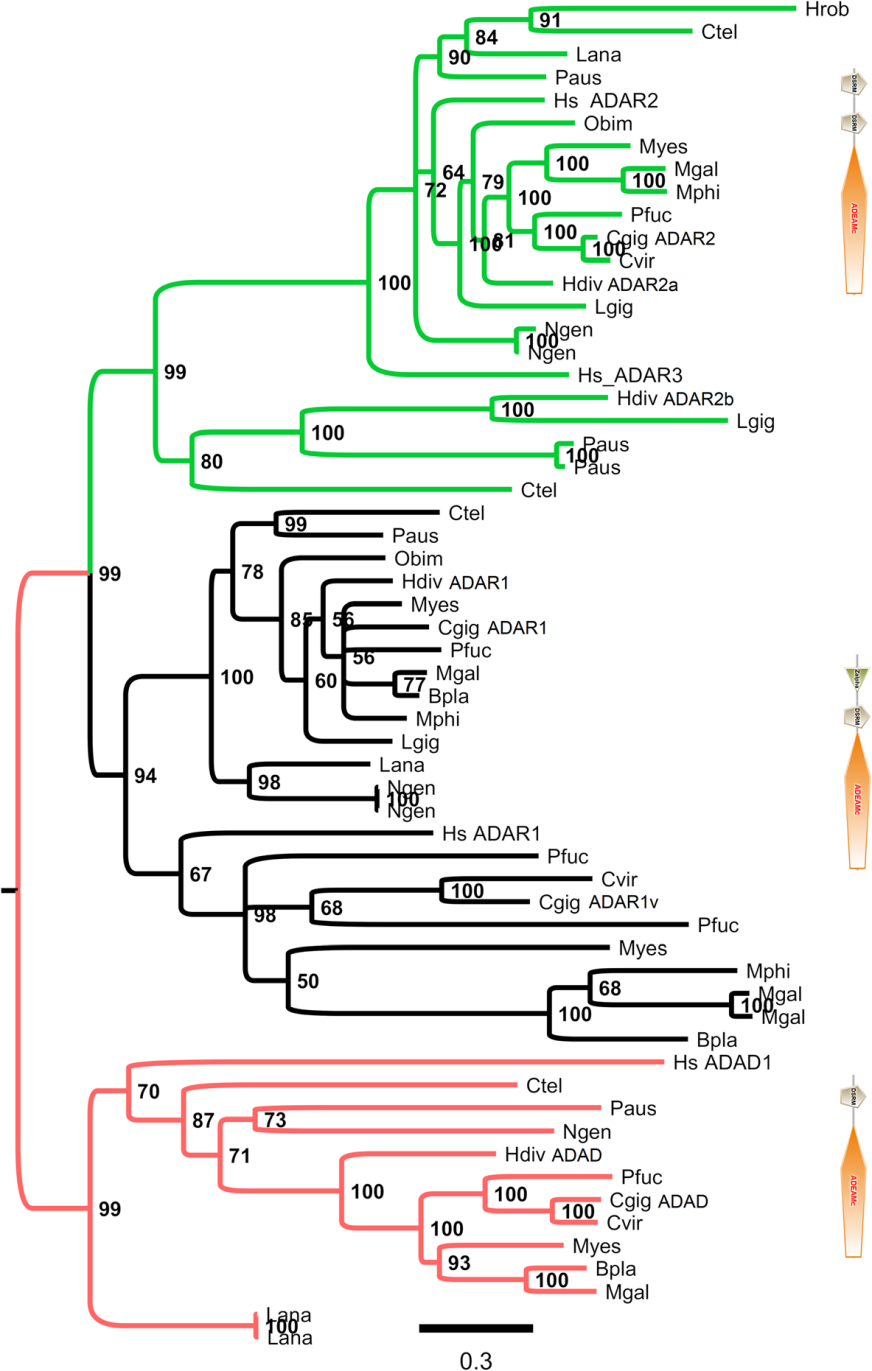
Bayesian phylogenesis of ADAR proteins from several lophotrochozoans and *Homo sapiens* (Hs). The phylogenetic tree is based on the multiple alignment of the catalytic domain provided as Additional file 1. Green, black and red colors denote the ADAR2, ADAR1 and ADAD clusters, respectively, while the typical domain construction of these proteins is reported on the right. Posterior probability values are indicated at each node. Lophotrochozoan species names were abbreviated to 4 letters (e.g. *Crassostrea gigas*, Cgig)

We identified four *ADAR-like* sequences by searching the *adenosine deaminase* domain in the *C. gigas* gene models. The corresponding protein sequences, EKC20855, EKC29721, EKC32699 and EKC39025, combined the conserved *adenosine deaminase* domain with a variable number of DNA and/or RNA binding domains (*Z-alpha* and *dsrm* domains, respectively). Running similar searches in 14 lophotrochozoan genomes, including species from the phyla of Mollusca (9), Annelida (2), Brachiopoda (2) and Nemertea (1), and in one transcriptome assembly (1 gastropod) we identified 48 *ADAR-like* genes (Table 1). Since no genome is available for the gastropod *H. diversicolor supertexta*, we reconstructed its transcriptome using RNA-seq data [68] and, among the predicted genes, we identified 4 *ADAR-like* transcripts. Moreover, all the analyzed genomes encoded a variable number of proteins characterized by the *adenosine deaminase* domain alone and to be regarded as candidate homologs of the ancestral ADAT gene. According to the nomenclature of vertebrate ADARs, which is based on the number of DNA and RNA binding domains and on the global protein architecture, we could preliminary classify the 48 lophotrochozoan ADARs as ADAD (one RNA binding domain), ADAR2 (2 RNA binding domains) or ADAR1 (at least one DNA and one RNA binding domains) (Additional file 2: Figure S1). Bayesian phylogenesis performed on the enzymatic domain of lophotrochozoan and *Homo sapiens* ADAR proteins resulted in three main clades reflecting the ADAR2, ADAR1 and ADAD gene classification (Fig. 1). While ADAD and ADAR2 are single copy genes in most of the analyzed organisms, the ADAR1 sequences clustered in two separate subclades because of the presence of two ADAR1 genes in the bivalve species. Exceptions were represented by the *C. virginica* genome which encodes one ADAR1 gene only and by the gastropods *Lottia gigantea* and *H. diversicolor supertexta* whose two ADAR sequences clustered into the ADAR2 clade.

**Table 1:** Lophotrochozoan ADARs identified in datasets representing 14 genomes and 1 transcriptome. Species name, phylum, origin and number of the identified ADAR sequences are reported

Species name	Phylum	Sequence origin	ADAR sequences
*Crassostrea gigas*	Mollusca	Gene models	4
*Crassostrea virginica*	Mollusca	Gene models	4
*Pinctada fucata*	Mollusca	Gene models	4
*Mytilus galloprovincialis*	Mollusca	Gene models	5
*Bathymodiolus platifrons*	Mollusca	Gene models	3
*Modiolus philippinarum*	Mollusca	Gene models	3
*Mizuhopecten yessoensis*	Mollusca	Gene models	4
*Lottia gigantea*	Mollusca	Gene models	3
*Octopus bimaculoides*	Mollusca	Gene models	2
*Phoronis australis*	Brachiopoda	Gene models	5
*Lingula anatina*	Brachiopoda	Gene models	4
*Notospermus geniculatus*	Nemertea	Gene models	5
*Capitella teleta*	Annelida	Gene models	4
*Helobdella robusta*	Annelida	Gene models	1
*Haliotis diversicolor*	Mollusca	Transcriptome	4

**Fig. 2:**
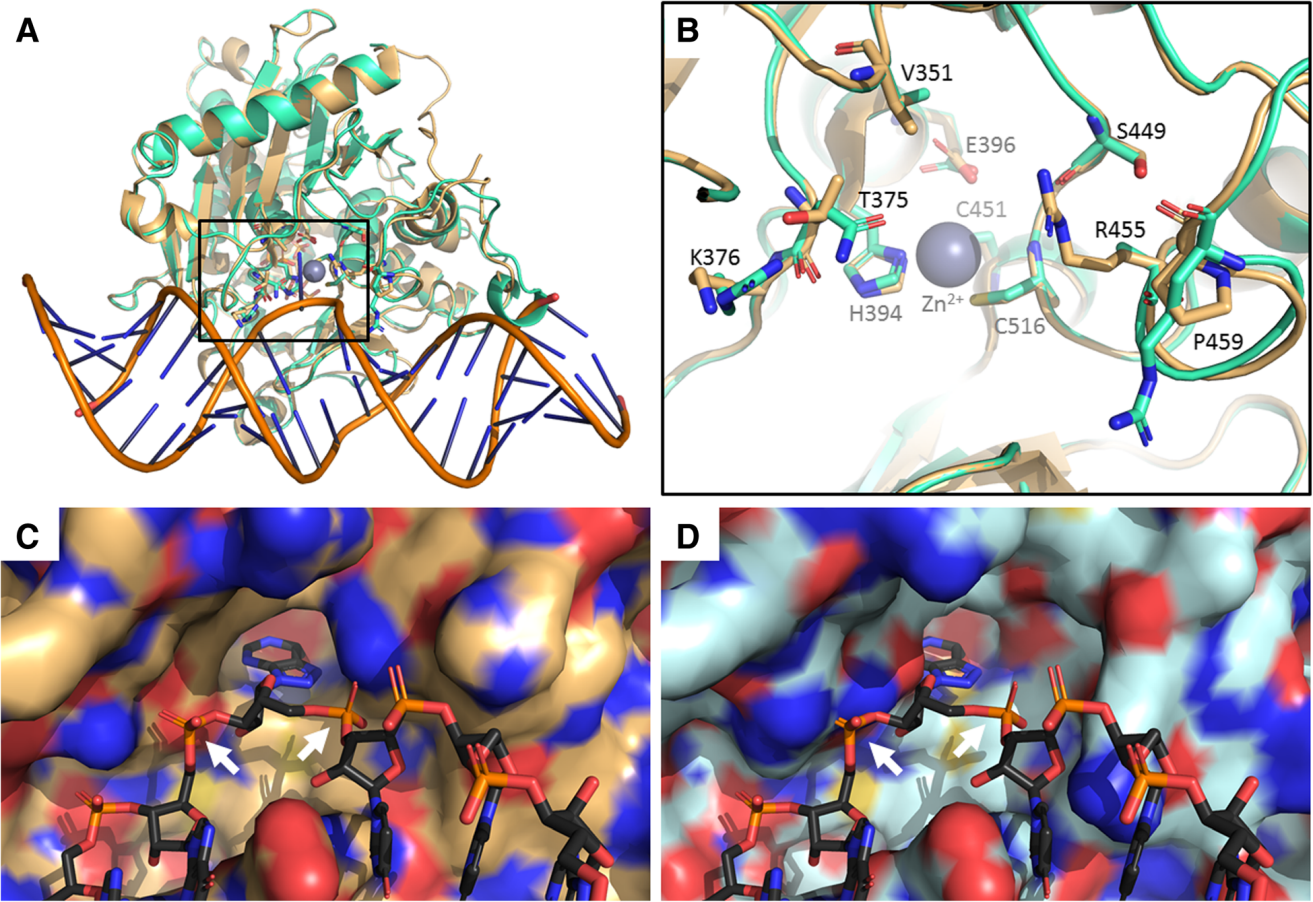
In silico structure model of the CgADAR1 deamination domain. **a**. Superimposition of the modeled structure of CgADAR1 (green cartoon) to the template HsADAR2 (pale orange cartoon) in complex with dsRNA (PDB ID: 5HP3). The active sites with the RNA flipped base are framed into the black box. **b**. Magnification of the superimposed active sites. Important residues for the protein’s activity and dsRNA binding, and mutated ones in ADAR1s respect to ADAR2s are represented in sticks. Surface representation of HsADAR2 (**c**) and CgADAR1 (**d**) active sites in the dsRNA bound state (represented in dark gray sticks). White arrows indicate the RNA phosphate groups (3′ left, 5′ right) of the flipped, deaminated base, anchored to conserved HsADAR2 residues which are mutated in CgADAR1. Worthy of note is the different steric hindrance and charge distribution of the active sites due to these mutations

Using the *C. gigas* ADAR1 (EKC29721) sequence as a query to identify similar structures in the Protein Data Bank (PDB) database, we initially recognized the most similar templates for the different domains. However, none of the feasible PDB structures did cover the full-length sequence of any ADAR family member. Only the C-terminal catalytic domain of the human ADAR2 isoform has been studied from a structural and functional point view for its implication in neurological disorders and cancer, whereas the same deaminase region of ADAR1 lacks of structural information [69, 70]. The dsRNA binding motifs as well as the Z-domains present in the N-terminal region of human ADAR1 and ADAR2 have been characterized by NMR techniques or X-Ray crystallography [71–73]. Given the highly conserved fold of the catalytic domain devoted to RNA binding and deamination, we decided to focus our attention on the catalytic domain only. The 364 residues (His 298 to Leu 671; numbering system in accordance with the ADAR2 structure) of the C-terminal catalytic domain region of the *C. gigas* ADAR1 have been used in the analysis. The search for evolutionary related structures allowed us to identify the ADAR2 catalytic domain structure as the best template (38.5% sequence identity) both in the apo form (PDB ID: 1ZY7) and in complex with RNA duplexes mimicking the reaction intermediate by a deaminated adenine (8-azanebularine (N) hydrate) flipped and bound to Zinc in the active site (PDB IDs:5ED2, 5ED1, 5HP2, 5HP3 [70]). Subsequently, the *C. gigas* ADAR1 model was compared with the ADAR2 template in complex with RNA duplex (Fig. 2). Given the robust sequence identity and overall reliability of the model, the catalytic domain fold and major structural features resulted to be highly conserved, as expected. Key residues involved in the coordination of zinc were all present in the active site (Cys451, Cys516 and His394), as well as the glutamate side chain involved in the catalysis as proton donor and Lys483, that stabilizes residues directly engaged in the metal coordination. Following homology modeling, we could highlight features unique to the ADAR1 enzymes (Table 2). Strikingly, the highly conserved ADAR2 Arginine 455 is mutated into Alanine (Ala455) in oyster ADAR1, while Arg376 is maintained. In human ADAR2, these two arginines establish symmetrical interactions with 5′ and 3′ phosphate groups upstream and downstream the flipped base, anchoring the latter in the proper orientation for catalysis. The Arg455 to Ala mutation breaks such symmetry, reduces the steric hindrance on one side and could weaken the substrate binding. A compensatory role can be attributed to the replacement of ADAR2 Pro459 with Arginine in *C. gigas* ADAR1 (Lys in human ADAR1), located in an adjacent loop (Fig. 2b). A role of the latter residue in substrate binding and proper orientation could be supposed either by forming H-bonds with the sugar backbone or 5′ phosphate binding. If this is the case, the phosphate group is coordinated with a different geometry by Arginine or Lysine side chain assuming the proper orientation by exploring one of its favored rotamers. However, an analogous role cannot be hypothesized in *H. diversicolor* supertexta devoid of the only Arg455 residue and showing conservation of Pro459. The entire loop from 454 to 477 residues, including Arg to Ala455 and Pro to Arg459 variations, can undergo a significant rearrangement upon binding of RNA duplex in ADAR2 and mutations in this region define the most significant features that distinguish ADAR1 enzymes from ADAR2. Furthermore, the amino acids shaping the binding site at the 3′ side of the flipped edited base maintained the same charge and hydrophobicity properties but were bulkier in all the ADAR1 enzymes analyzed, where the couple of residues Asn375 and Arg376 replaced the Thr375 and Lys residues of ADAR2 (Fig. 2c). Altogether, the variations observed in ADAR1 enlarge the active site region involved in the coordination of the RNA substrate at the 5′- phosphodiester flanking region of the flipped base and reduce the accessible space at the 3′-phosphodiester side, causing obliged repositioning of substrates for a productive geometry and putative changes in the enzyme selectivity and catalytic parameters (Fig. 2d, Additional file 7).

**Table 2:** Functionally relevant aminoacid variations between human, oyster and abalone ADAR proteins

Organism	ADAR ID	Residue position					
		351	375	376	449	455	495
*H. sapiens*	ADAR2	Val	Thr	Lys	Ser	Arg	Pro
	ADAR1	Val	Asn	Arg	Ala	Ala	Lys
*C. gigas*	ADAR2	Val	Thr	Lys	Ser	Arg	Pro
	ADAR1v	Val	Asn	Arg	Ala	Ala	Arg
	ADAR1	Val	Asn	Arg	Ala	Ala	Arg
*H. diversicolor*	ADAR2a	Ile	Asn	Arg	Pro	Ser	Thr
	ADAR2b	Val	Thr	Lys	Ala	Arg	Pro
	ADAR1	Ile	Asn	Arg	Ala	Ala	Pro

positions are based on HsADAR2 (Matthews et al., 2016, ref. [70])

### Expression patterns of *C. gigas* ADAR genes

One of the oyster ADARs (identified as GCI_10012998 or EKC29721 and herein classified as ADAR1) was previously found upregulated in a time-course infection experiment with the dsDNA virus OsHV-1 [20], as well as in a single sample of oyster spat naturally infected by the same virus [17]. After these first studies, the expression of the second ADAR1 paralog (EKC20855) has been investigated by qRT-PCR and reported as mildly regulated during OsHV-1 infection [74]. We analyzed the expression profiles of the four *C. gigas* ADAR genes in available RNA-seq samples obtained from oyster naïve tissues, developmental stages, abiotic stimuli, bacterial challenges, and viral infections (Additional file 8). In addition to the available datasets, we sequenced the total RNA of one oyster sample, selected in a batch of oysters deployed in the lagoon of Goro in a critical seasonal period (see Methods). In addition to oyster transcriptome analysis, we computed the number of reads mapping on the OsHV-1 genome in parallel, in order to measure the transcriptional activity of the virus.

RNA-seq data analysis demonstrated that oyster ADAR genes are most often expressed at low, but still detectable, levels (Additional file 9 and Additional file 3: Figure S2). One oyster ADAR1 (EKC29721, hereinafter renamed CgADAR1v) displayed higher peak values of expression compared to those of the other ADARs (reaching 795 versus 25–54 TPMs). The expression profile of CgADAR1v well-matched the number of viral reads in the same samples. Basically, CgADAR1v showed highest expression values in the few samples showing abundant OsHV-1 reads: one oyster spat sample named G1 [17], samples referring to laboratory infection trials [23] and in few developmental stages of oysters infected with OsHV-1 [75] (Additional file 9 and Additional file 3: Figure S2). More specifically, the CgADAR1v expression followed the amount of OsHV-1 reads over a 0–72 h post injection (hpi) in an infection trial performed with both resistant and susceptible oyster families. Notably, the basal expression of CgADAR1v and its expression up to 12 hpi in the resistant oyster family was twice the value observed in susceptible oysters, and the CgADAR1v expression dramatically increased starting from 24 hpi in the susceptible oyster family (Additional file 9 and Additional file 3 Figure S2). We also observed a relatively high CgADAR1v expression in three oyster samples negative for OsHV-1, but including RNA virus sequences [6] (37–70 TPMs Additional file 9). Apart from these samples, the expression levels of CgADAR1v were very limited (median of 9.6 TPM over 202 RNA-seq samples). Contrary to CgADAR1v, the expression of CgADAR2 (EKC32699) and of the second CgADAR1 gene (EKC20855) was always detectable, whereas the expression of CgADAD (EKC39025) was evident only in the first stages of oyster ontogeny (Additional file 9 and Additional file 3: Figure S2).

**Fig. 3:**
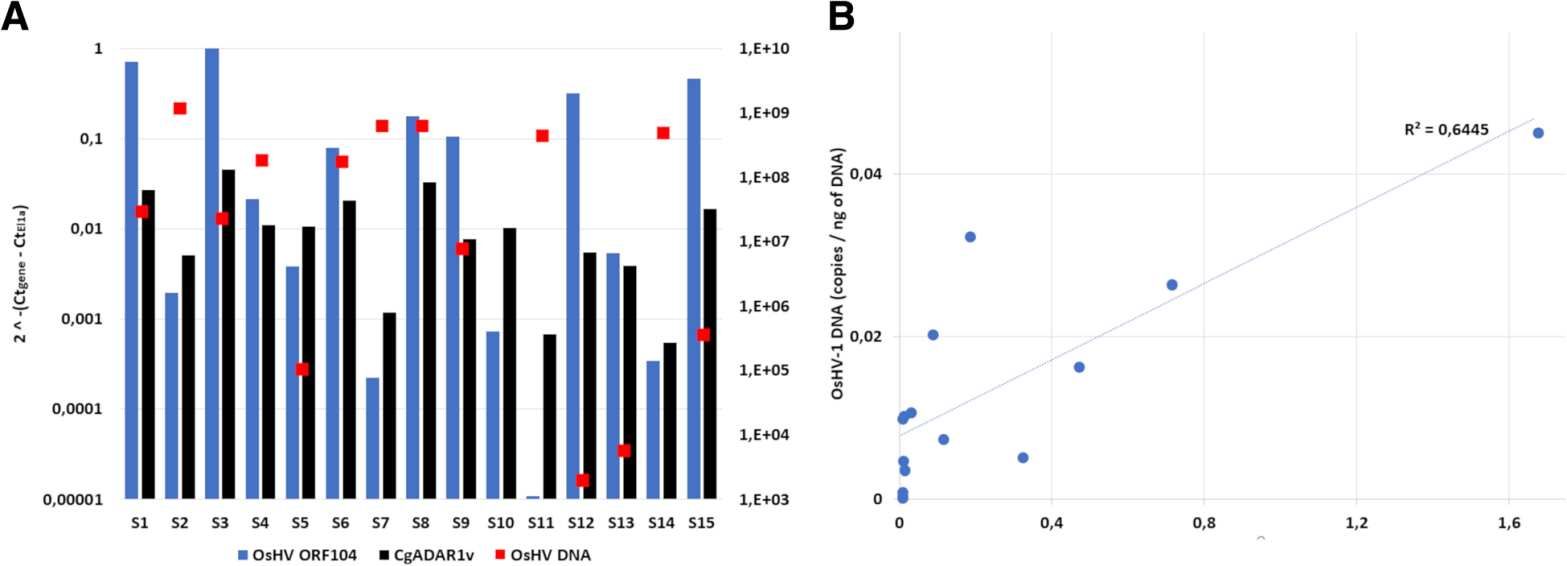
**a**. Expression values of OsHV-1 ORF104 (blue) and CgADAR1v (black) in 15 individual oysters (S1-S15) deployed in the lagoon of Goro (North Adriatic Sea, Italy, 2016). qRT-PCR expression data were normalized against CgEl1α. The OsHV-1 DNA copies per ng of total DNA (red points) detected in the same samples are also reported in log_10_ scale (values on the secondary Y axis). **b**. Correlation between OsHV-1 ORF104 and CgADAR1v expression values

In addition to RNA-seq data analysis, we individually measured the expression of CgADAR1v in 15 oysters showing variable amounts of OsHV-1 DNA. To estimate the OsHV-1 transcription in these samples, we measured the expression of OsHV-1 ORF104, a gene tentatively annotated as *major capsid protein* [16] and used as a proxy of overall viral transcription according to previous analysis [76]. qRT-PCR data revealed a certain correlation (R^2^ = 0.65) between the expression of CgADAR1v and of OsHV-1 ORF104 (Fig. 3). CgADAR1v expression levels ranged from 4.5% of the expression of the housekeeping gene *Elongation factor 1-alpha* (El1α), in the S3 sample characterized by the highest level of ORF104, to 0.06% in the S11 sample with the lowest ORF104 expression level.

### Expression analysis of *Haliotis diversicolor supertexta* ADARs

**Fig. 4:**
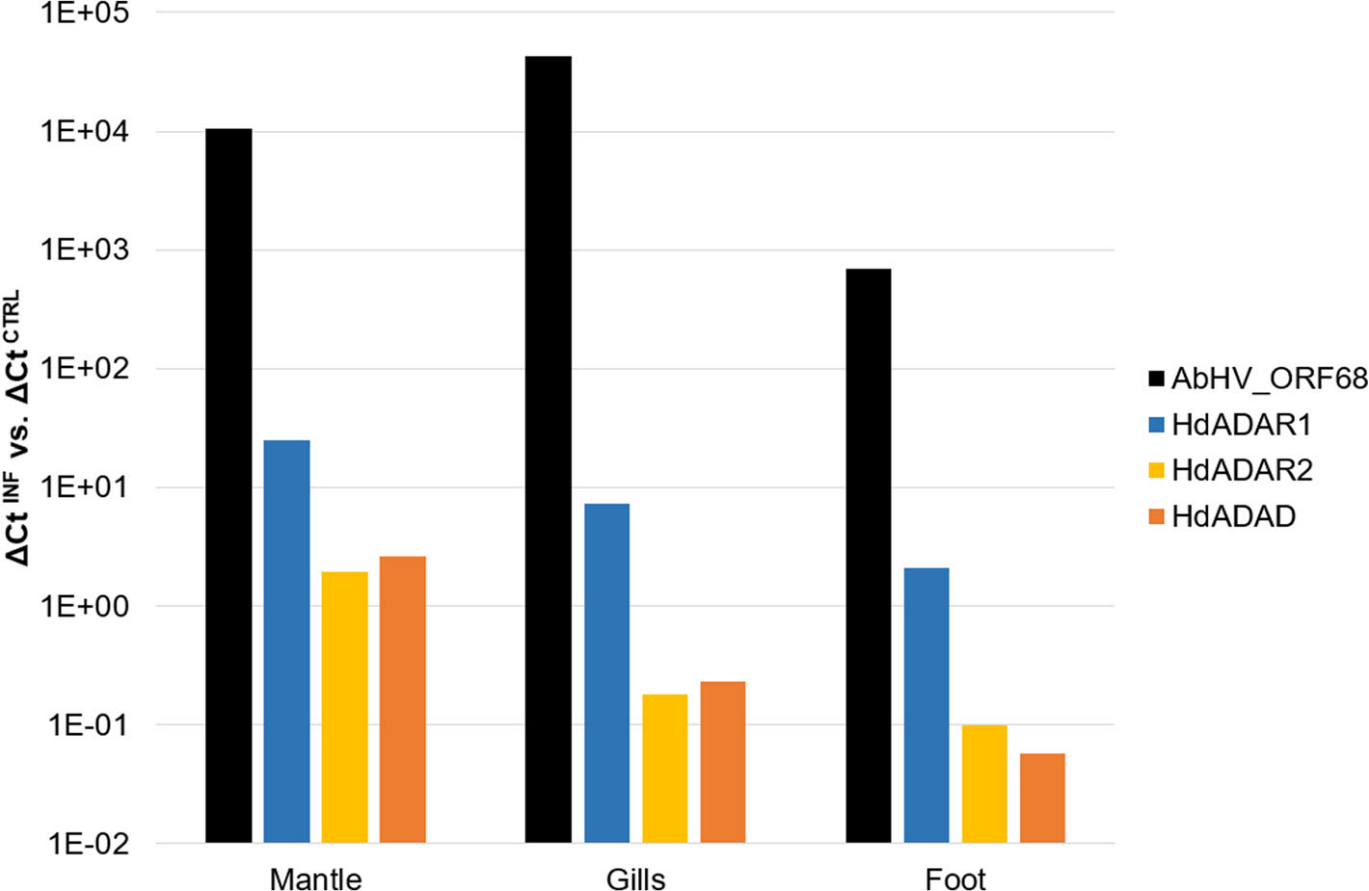
qRT-PCR expression analysis of *Haliotis diversicolor supertexta* ADARs. For AbHV-1 ORF68, HdADAR1, HdADAR2 and HdADAD the ratio in log_10_ scale of the deltaCt between viral infected and control samples are reported for the mantle, gill and foot tissues of one abalone (M49)

Contrary to *C. gigas*, the expression of the four ADAR genes in *H. diversicolor supertexta* (HdADARs) was never tested before. We exploited the RNA-seq data from a time course study (0, 24 and 60 hpi, 3 biological replicates per time point) performed on abalones infected with AbHV-1 to investigate the expression profiles of HdADARs [68]. As for *C. gigas*, RNA-seq data revealed an overall very limited expression of HdADARs. Nonetheless, HdADAR1 showed a fair level of expression levels in two of the three abalones sampled at 60 hpi and in one abalone at 24 hpi, although with considerably lower levels compared to oyster (Additional file 9). To verify these results, we analyzed by qRT-PCR the expression levels of HdADARs in three selected tissues (mantle, gills and foot) at 0 hpi (3 abalones samples) and 60 hpi (1 abalone, sample MA49). To estimate the AbHV-1 transcriptional activity we measured the expression of AbHV-1 ORF68, identified as the homolog of OsHV-1 ORF104. Overall, we obtained reliable qRT-PCR data for three out of four HdADARs, since one ADAR2 paralog (HdADAR2b) showed negligible and poorly reproducible values. As for *C. gigas*, and partially confirming RNA-seq data, the gene classified as ADAR1 was considerably upregulated in the viral infected sample at 60 hpi (Fig. 4). HdADAR1 was mostly induced in mantle (24x) with lower induction levels in gills and foot (7x and 2x, respectively). The other two HdADAR genes were stably expressed in mantle while they were under-regulated in gills and foot (10x and 20x for HdADAR2 and HdADAD, respectively).

### Study of ADAR1 during viral infection

To evaluate the functional role of ADAR1 during in vivo Malacoherpesvirus infection, we analyzed paired DNA- and RNA-HTS data originated from infected oysters and abalones. At present, data of this sort are available only for a Chinese *Haliotid herpesvirus-1* infecting abalones (called AbHV-1-CN2003, Table 3). To expand the dataset with a second case study, we sequenced the whole RNA of a single oyster (sample S15, see Fig. 3, Additional file 3: Figure S2 and Additional file 9) obtained from the same geographical area from where we recently sequenced the OsHV-1-PT genome [77], using a ribo-depletion approach in order to minimize the possible 3′-read bias associated to polyA-selection procedure and to capture also possible non-polyadenylated RNAs. We selected the S15 sample because it was characterized by intermediate amount of OsHV-1 DNA (3.7 × 10^5^ copies/ng of total DNA) and significant expression levels of both CgADAR1v and OsHV-1 ORF104 (Fig. 3a). Illumina sequencing yielded 54.1 million of high-quality reads, pertaining to *C. gigas* (45%) or OsHV-1 (2.05%) according to their mapping on the corresponding genomes. RNA-seq analysis confirmed both the massive OsHV-1 ORF transcription and the activation of oyster antiviral pathways, i.e. the expression of several genes previously described as upregulated during OsHV-1 infection [17]. In agreement with the qRT-PCR results, CgADAR1v was considerably expressed in the S15 sample (103 TPMs, Additional file 9).

**Table 3:** Genome sequence data available for Malacoherpesviruses. Virus name, variant acronym, reference paper and availability of DNA- and RNA-seq data are reported. The cases with paired RNA/DNA HT-data are underlined

Virus name	Variant ID	Reference	HT-DNA data	HT-RNA data
OsHV-1	Reference 2005	(Davison et al., 2005) [10]	N	N
	France μvar	(Burioli et al., 2017) [24]	na	N
	Ireland μvar	(Burioli et al., 2017) [24]	na	N
	Italy μvar	(Abbadi et al., 2018) [77]	Y	Y
	OsHV-1-SB	(Xia et al., 2015) [104]	N	Y
	AVNV	(Ren et al., 2013) [105]	N	N
AbHV-1	Victoria	(Savin et al., 2010) [106]	N	N
	Taiwan	KU09699.1	N	N
	AbHV-1-CN2003	(Bai et al., 2019) [78]	Y	Y

Y: sequencing carried out with HTS instruments and data available; N: non-HTS datasets (genomes obtained with Sanger technology or not used for RNA-seq experiments); na: HTS data not available

**Fig. 5:**
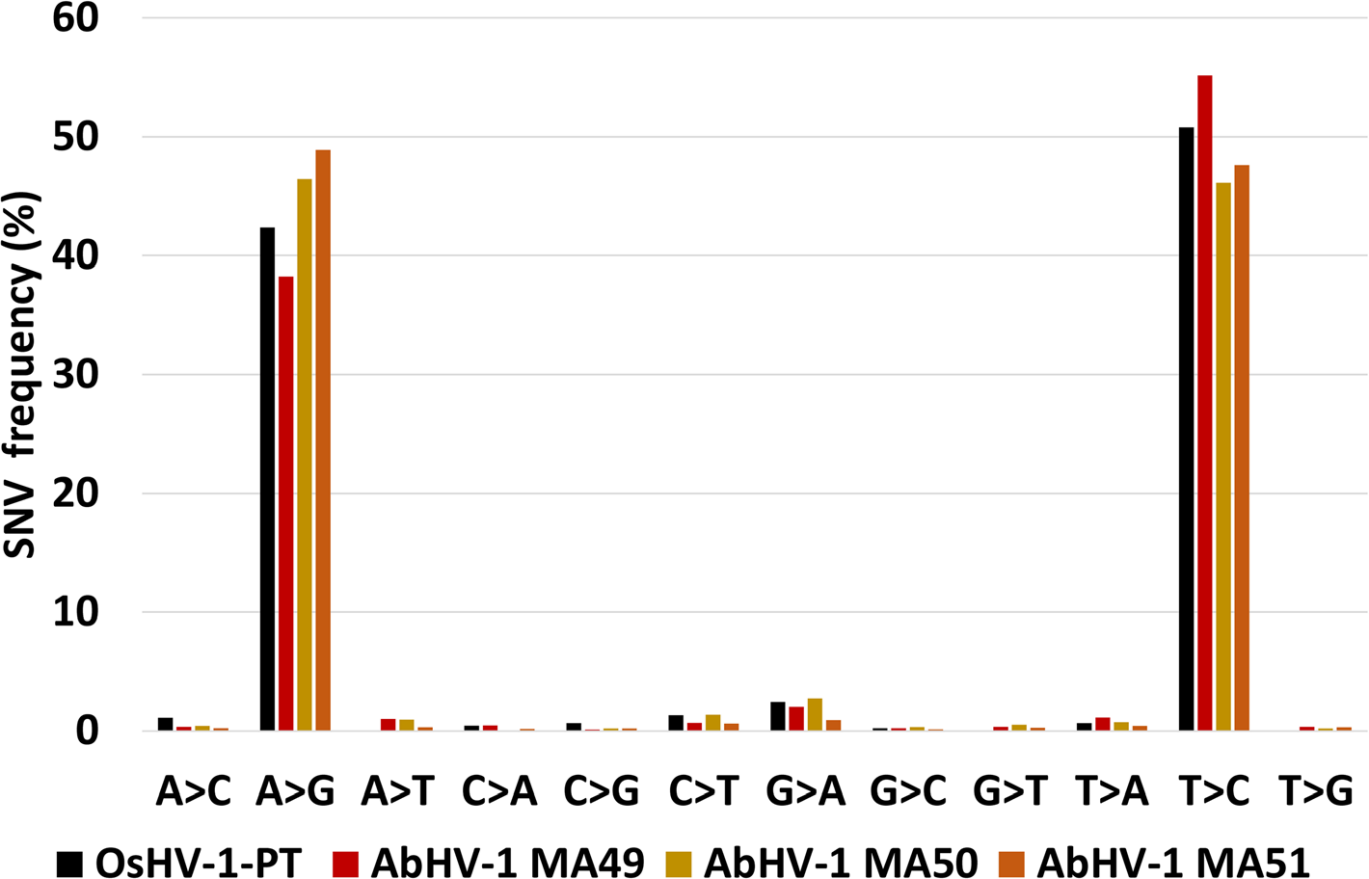
Sequence nucleotide variation (SNV) profiles detected in the viral RNAs obtained from Malacoherpesviridae-infected mollusks. Relative frequency of each possible sequence change based on OsHV-1-PT RNA (one oyster sample, black) and AbHV-1 RNA (three abalone samples collected at 60 hpi; red, yellow and orange)

We exploited the paired DNA- and RNA-HTS data from abalone and oyster to analyze the Single Nucleotide Variation (SNV) profiles obtained by mapping each set of viral RNA-seq reads on the corresponding virus genome, namely AbHV-1-CN2003 and OsHV-1-PT. The AbHV-1-CN2003 RNA- and DNA-seq data have been generated from two comparable biological samples, i.e. the viral homogenate used for the experimental infection (DNA-seq) and individual abalones sampled at 24 and 60 hpi (RNA-seq [68, 78]). Instead, the OsHV-1-PT RNA- (S15) and DNA-seq data were obtained from two distinct samples from the same geographical area where OsHV-1 was recurrently detected. To limit the potential bias of analyzing nucleotide variations from heterogeneous DNA and RNA datasets, we removed 54 SNVs occurring with a high frequency (> 95%, Table 4) in the OsHV-1 RNA sample, assuming that they arose from genome-encoded variations. According to the homogenous DNA and RNA samples, no such high-frequency variation was found among AbHV-1-CN2003 RNA SNVs. SNV calling identified 5,849 different RNA SNVs in the abalone samples and 451 SNVs in the OsHV-1-PT sample, with a median SNV frequency per sample of 1.5–1.7% for the AbHV-1-CN2003 samples at 60 hpi and 1.7% for the S15 sample, whereas we observed an higher SNV frequency for the AbHV-1-CN2003 samples at 24 hpi (Table 4). We showed that most of the SNVs are A-to-G or T-to-C variations, 64–97% of the AbHV-1-CN2003 SNVs and 94% of the OsHV-1-PT SNVs (Table 4, Fig. 5), suggesting an ADAR-mediated editing of RNAs for the transcriptional products of both viruses. A-to-G and T-to-C variations occur as genomic hotspots in both viral genomes (Additional file 4: Figure S3, panels A and C), although the most targeted viral genes are not conserved between OsHV-1 and AbHV-1. We retrieved the highest number of SNV (2,773) in the sample with the highest number of viral reads, a fact suggesting that ADAR-editing is dependent upon the quantity of viral RNA (Table 4). Although AbHV-1-CN2003 ADAR-compatible SNV occurred at hotspots in the viral genome (Additional file 4: Figure S3C), SNVs appeared differently distributed among samples, with most of them (72%) occurring only in one sample.

**Table 4:** Single Nucleotide Variations identified in AbHV-1-CN2003 OsHV-1-PT RNA data. The number of viral reads in millions, the total number of SNV, the number of genomic encoded SNVs and of ADAR-compatible SNV are reported for six abalone samples and two oyster samples (sample S15 and, for comparison, one pooled sample of oyster spat having similar geographical origin and collected in 2011)

Virus	Sample ID	No. of viral reads [M]	No. of SNV	No. of genomic SNVs	No. of ADAR SNV	% of ADAR-SNV	Frequency of ADAR SNV
AbHV-1-CN2003	24 hpi	0.6	2,645	0	2,466	93	5.8
	24 hpi	0.2	296	0	222	75	12.3
	24 hpi	0.03	48	0	31	64	17.4
	60 hpi	4.3	889	0	836	94	2.7
	60 hpi	3.6	856	0	808	94	1.4
	60 hpi	5.8	2,847	0	2,773	97	1.5
OsHV-1-PT	S15	1.1	505	54	423	94	1.7
	G1	3.3	463	48	297	72	1.6

The identification of numerous T-to-C substitutions was generally ascribed to the non-specific directionality of RNA-seq reads when mapped on a genomic reference [79], meaning that they could represent A-to-G variations reported on the reverse strand. Since we used a stranded library to sequence the whole RNA of the S15 sample, this SNV distribution was unexpected because these reads should retain the strand-information and map only on the coding strand. To further investigate this point, we directionally-mapped the reads on the OsHV-1 genome, according to the strand direction suggested by the annotated viral genes. As a result, 1,062,559 reads mapped according to the coding direction, whereas 130,111 reads mapped on the opposite strand (R and F reads, respectively, in Additional file 4: Figure S3, panel A). Most of the reversely mapped reads (F reads) flanked viral genes with similar orientation, suggesting that the F reads originated from 5′ or 3′ UTRs, which are located in genomic regions overlapping antisense ORFs (Additional file 5: Figure S4, panel A). SNV detection performed separately on R and F reads demonstrated that R-SNVs were mostly A-to-G, whereas F-SNVs were quite exclusively T-to-C (Additional file 4: Figure S3, panel B). These results indicate that most of the T-to-C SNVs represent genuine A-to-G variations located on 5′ or 3′ UTRs of a given gene whose overlap a second gene located on the opposite strand (Additional file 5: Figure S4, panel A). Moreover, we identified some archetypical situations supporting the existence of SNV hotspots in the OsHV-1 genome (Additional file 5: Figure S4, panels B to F).

### Target preferences of ADAR1

**Fig. 6:**
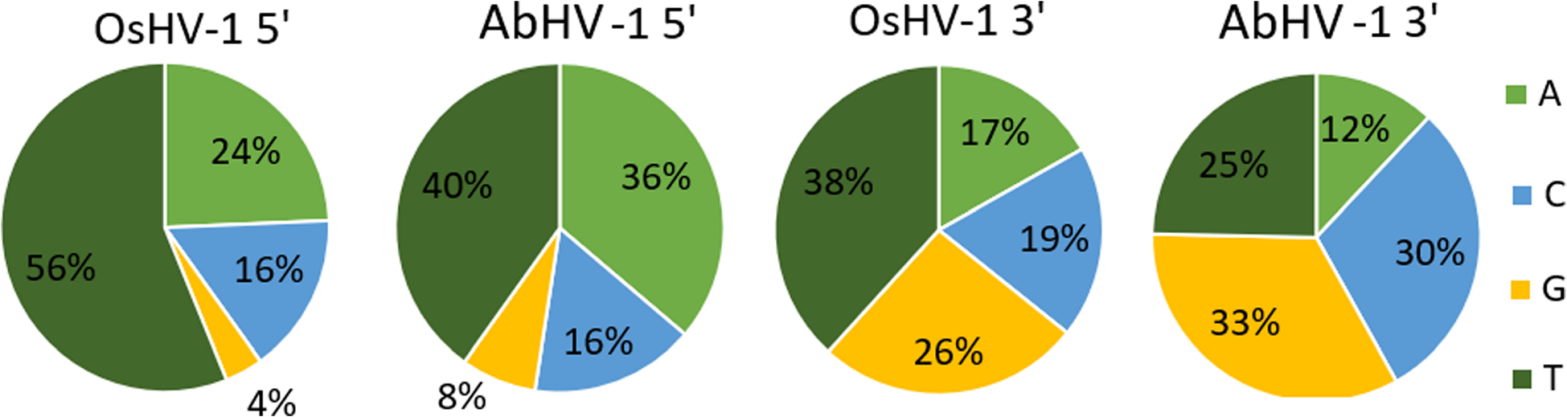
Frequency of different nucleotides (A, C, G, T) at the 5′ and 3′ positions flanking the ADAR-edited base (A) in OsHV-1 and AbHV-1 genomes

To investigate if oyster and abalone ADAR1s edited preferential adenosines among the ones of the viral genomes, we mapped the nucleotide distribution of the upstream and downstream positions flanking ADAR-compatible SNVs. For both viral genomes, ADAR-SNVs mostly occurred on nucleotides with thymine, adenine or cytosine in the upstream position, whereas downstream positions did not show a strong base preference (Fig. 6). This result confirmed that TA/CA and AA are strong ADAR targets, compared to the GA dinucleotide motif which is reported as a weak target [55, 61].

### SNV profile of the *C. gigas* transcriptome

We compared the distribution of the OsHV-1 SNV types with the distribution of *C. gigas* SNVs. We mapped the S15 reads on the oyster genome and we called 404,508 SNVs, partly referring to high-frequency SNVs (genome-encoded) as expected by the heterogenicity between the genome of the Italian oyster and the reference Chinese one [80]. After removing 75,223 of such SNVs, 209,620 of the remaining SNVs resulted to be located in protein coding regions and were retained for subsequent analysis. Since the oyster genome is not annotated with UTRs, we *de-novo* assembled the S15 reads and we back mapped the reads on the mRNA contigs annotated with CDS and UTRs, as well as on putative non-coding RNAs (ncRNAs). The SNV calling algorithm predicted 274,604 SNVs on mRNA, whose 66% are located on CDSs and 34% on UTRs, and 173,387 SNVs on putative ncRNAs. Analyzing the frequencies of the different types of substitutions, we could not identify a preferential SNV type, nor for coding SNVs, neither for UTR ones nor for SNVs occurring on ncRNAs (Additional file 6: Figure S5).

### Overview of the functional activity of *C. gigas* ADAR on available RNA-seq data

**Fig. 7:**
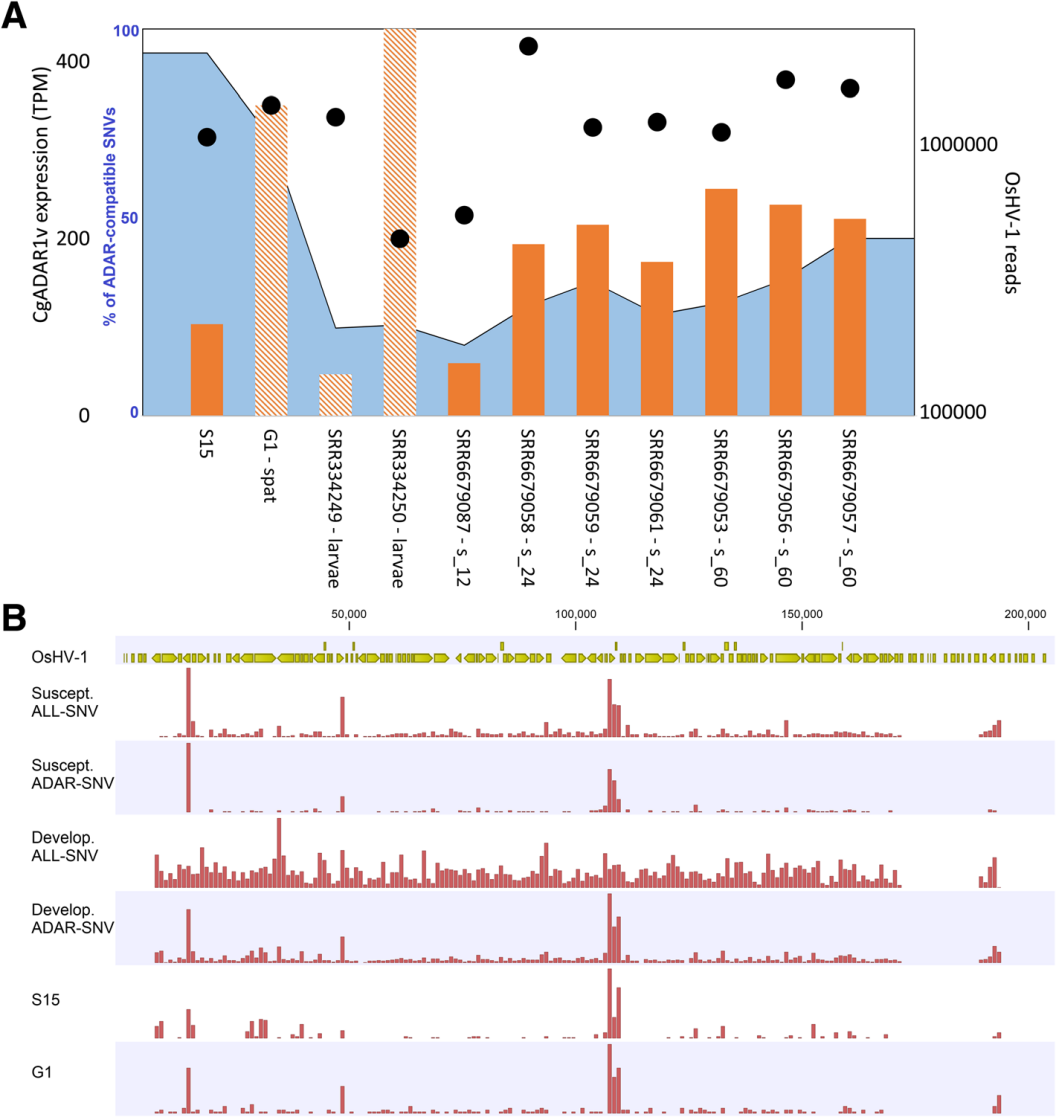
ADAR editing of OsHV-1 RNA. **a**. The expression levels of CgADAR1v (TPM, orange bars), the number of OsHV-1 reads (secondary Y-axis in log scale, black circles) and the percentage of ADAR-compatible SNVs over the total number of detected SNVs (blue area depicted on 0–100 scale) are reported for 11 virus-infected oyster samples showing at least 200,000 viral reads. The samples pertaining to pooled individual are depicted by dashed bars. **b**. Hotspots of ADAR1 editing sites as mapped on the OsHV-1 genome. Viral ORFs and their coding directionality are in yellow. The genome distribution of total and ADAR-compatible SNVs are reported for the susceptible oyster family (12, 24 and 60 hpi samples) and for the developmental stages (two samples). For comparison, the distribution of ADAR-compatible SNVs were also reported for the S15 and G1 samples

Although the main objective of this work was to exploit paired RNA and DNA-seq data to call low-frequency SNVs occurring on Malacoherpesvirus genomes, we aimed to provide a more extended overview of ADAR activity during OsHV-1 infection by comparing available datasets. We selected, among the available *C. gigas* RNA-seq samples, the ones with enough viral reads (setting an arbitrary cut-off to 200,000 viral reads) to effectively map the low-frequency SNVs typical of ADAR activity. Accordingly, we compared 11 samples, showing levels of CgADAR1v in the range of 60–430 TPMs. SNV calling identified 1,735 ADAR-compatible variable positions (38–423 per sample), mostly occurring in the conserved genomic hotspots although, as reported for abalone samples, SNVs involved different nucleotides among different individuals. The frequency of ADAR-compatible SNV over the total SNV resulted to be variable among samples, from 97% for the previously described S15 sample, to 18% in one individual oyster at 12 hpi, while this percentage is increasing at 24 and 60 hpi points (Fig. 7, panel A). Notably, we observed the lower percentages of ADAR-compatible SNVs in the pooled samples included in this analysis, namely the G1 and other developmental stages. Although other samples presented lower percentages compared with the S15 samples, the location of the ADAR-compatible SNVs was conserved and it was mostly confined in few hotspots along the OsHV-1 genome (Fig. 7, panel B).

### ADAR footprint in Malacoherpesvirus genomes

**Fig. 8:**
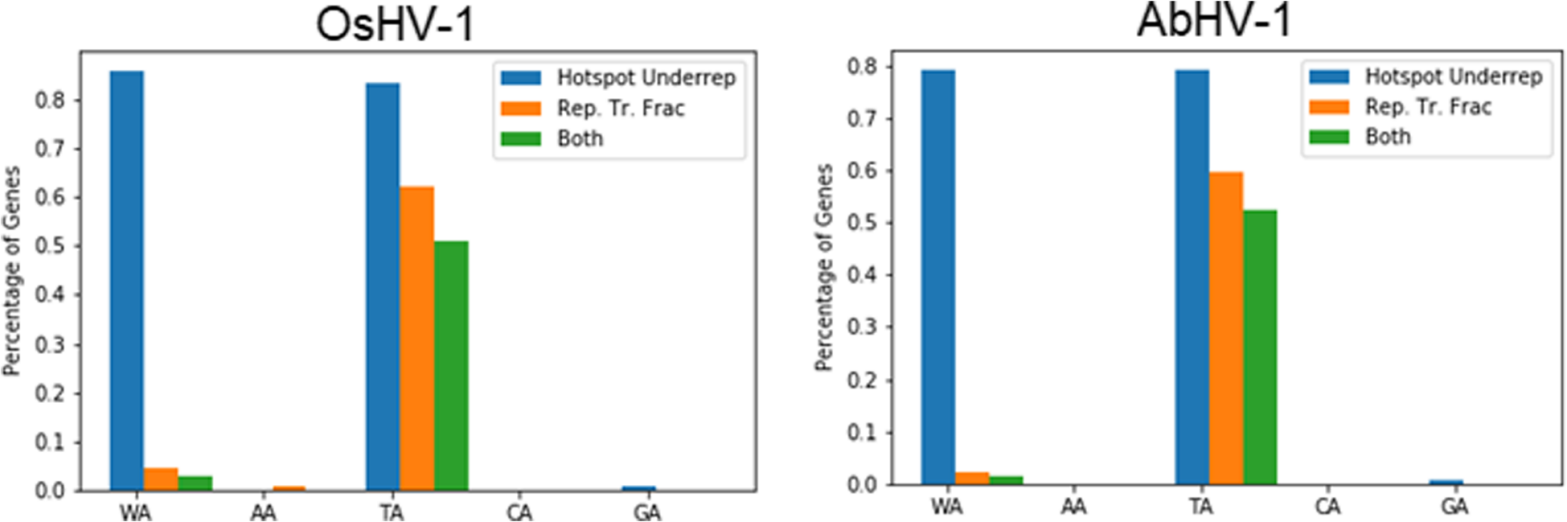
Under-representation and replacement transition fractions for OsHV-1 and AbHV-1. The blue bars denote the percentage of genes with an under-representation *P*-value < 0.05 (see Methods); the orange bars denote the percentage of genes with a replacement transition fraction (Rep. Tr. Frac.) P-value > 0.95; the green bars denote the percentage of genes with both the aforementioned *P*-values. All P-values were corrected using the Benjamini-Hochberg P-value adjustment

The analyses performed on oyster and abalone samples infected with the corresponding pathogenic Malacoherpesviruses demonstrated an intense ADAR editing targeting the transcription products of these dsDNA viruses. We wondered if this activity could result in an ADAR-footprint recognizable in the genomes of these viruses, meaning that, in the evolutionary time, these viruses have modified their DNAs to counteract the evolutionary pressure of ADAR activity. As reported elsewhere [79] and demonstrated in this study, the upstream neighbor nucleotide played an important role for the enzymatic activity of both CgADAR1v and HdADAR1. Based on this evidence, we used the CDUR package [81] to evaluate, for both malacoherpesvirus genomes, the frequencies of the GA, CA, TA, AA, and WA (W = A/T) dinucleotide motifs. Briefly, the program generates a null frequency distribution obtained from 1,000 shuffled genomes and compares this with the observed frequencies (see Methods section for further details). This analysis highlighted a strong under-representation of the TA motif in both the viral genomes, resulting in 83 and 79% of the OsHV-1 and AbHV-1 ORFs, respectively, with statistically-significantly fewer TA motifs then the null distribution (Fig. 8, blue bar). Our analysis demonstrated also that 51 and 52% of OsHV-1 and AbHV-1 ORFs maximized the TA under-representation, since, given an overall under-representation of TA hotspots, we found that most, if not all, of the remaining hotspots, if mutated, would alter the amino-acid sequence (Fig. 8, green bar). Accordingly, this analysis indicates the presence of a strong ADAR footprint in the genome of both viruses.

## Discussion

We exploited high-throughput RNA-sequencing data obtained from one bivalve and one gastropod species infected by OsHV-1 and AbHV-1, two Malacoherpesvirus variants detected in Italy and in China, respectively, to investigate the functional role of ADAR1 during virus infection. At present, ADAR-editing impacting dsDNA viruses has been reported only on target sequences, like structured lncRNAs [82] or miRNA precursors [67, 82, 83]. The evidence of ADAR self-editing in vertebrates [54] and in few, but important invertebrate species [58, 60, 79], suggests the evolutionary conservation of this post-transcriptional RNA editing process [79]. Conversely, the involvement of ADAR in the antiviral host defense was seldom reported in invertebrates [64, 66]. We present our work as first report of the functional activity of a lophotrochozoan ADAR1 in editing viral RNAs and as one of the few studies revealing an extensive ADAR1 hyper-editing of RNAs expressed by dsDNA viruses.

According to protein construction, phylogenetic analysis and structural modelling, we reported the conservation of ADAD, ADAR1 and ADAR2 gene types among most of the analyzed lophotrochozoan phyla (Fig. 1, Additional file 2: Figure S1 and Table 1) and, in particular, the existence of conserved structural features in metazoan ADAR1 proteins (Fig. 2). Although most of the analyzed lophotrochozoans encode several ADAR paralogs, we demonstrated that only one ADAR gene is upregulated during viral infection in the bivalve *C. gigas* and in the gastropod *H. diversicolor supertexta*, and this gene refers to an ADAR1 paralog (Figs. 3 and 4 and Additional file 3: Figure S2). The ADAR1 expression in *C. gigas* in tightly associated with the presence of OsHV-1 RNA, and, intriguingly, one oyster OsHV-1-resistant family showed higher basal ADAR1v levels compared to susceptible individuals (Additional file 3: Figure S2). Resembling the interferon pathway of vertebrates, we could suppose that viral nucleic acids are sensed by intracellular receptors allowing the activation of host’s antiviral defenses, including ADAR1 up-regulation. The existence and nature of such sensors as well as of the intermediate elements of signaling pathways have been only partially addressed so far and require functional proofs [32, 34].

When compared with ADAR2 proteins, the differences found in the residues near the enzymatic pocket of ADAR1 could underpin the different enzymatic efficiency and specificity of these two proteins. We suggested that, similarly to human ADAR1, CgADAR1v may be able to perform non-specific deamination of viral RNAs owing to a reduced site selectivity of the enzymatic pocket (Fig. 2). Accordingly, we observed a remarkable ADAR1 preference towards the nucleotide upstream the editing site of the enzyme, compared to the downstream position (Fig. 6). A similar evidence was reported following comparative analysis of the ADAR editome of invertebrate organisms [55].

The relevant ADAR1 over-expression in oyster and abalone resulted in extensive ADAR-mediated hyper-editing specifically impacting viral RNAs, as expected by the structural protein conservation (Fig. 5 and Additional file 4: Figure S3) and such situation is found also in most of the relevant RNA-seq dataset containing Malacoherpesvirus reads (Fig. 7). We want to point out that the methodology we applied to call SNVs is suitable to recover variations on abundantly transcribed (viral) genome regions, while it might be somewhat inappropriate to recognize ADAR-SNVs when referring to host sequences. In fact, ADAR editing mostly occurred in poorly expressed genome regions or in repeated elements [84] and more appropriate pipelines have been proposed to recover host hyper-edited reads from RNA-seq data [84]. Accordingly, we compared viral and host SNV profiles aiming to highlight that the activity of ADAR1 was mainly impacting viral RNAs, although we cannot exclude ADAR self-editing on specific target sites. ADAR hyper-editing emerged as a low frequency editing, typically occurring in genomic hotspots characterized by the presence of overlapping genes on the opposite strand (Additional files 4 and 5: Figs. 3 and 4). Although the length of viral UTR regions is still unknown, gene overlapping is a common condition in the dense coding genomes of these viruses (e.g. 84% of the OsHV-1 genome is predicted to be transcribed) and, moreover, these viruses are characterized by whole-genome transcription during the lytic stage [76]. Hence, we propose that both these conditions supported the functional role of ADAR1 in oyster and abalone. In fact, the co-transcription of viral genes which are encoded on the two DNA strands of partially overlapping genome regions will likely produce dsRNA, the typical ADAR substrate. Such evidence well correlated with the presence of SNV hotspots showing ADAR hyper-editing. Moreover, we got evidence of abundant T-to-C SNVs in regions showing gene overlap (Additional file 4: Figure S3), as reported for human T-to-C ADAR variations, with 66% of them found in regions with overlapping genes on opposite strands [85].

We also wondered if ADAR1 acts as pro- or as anti-viral protein against malacoherpesviruses. The existence of an extensive editing of viral UTRs seems to be in contrast with a strict antiviral role, which is expected to generate non-functional proteins by hypermutating viral transcripts [86]. We searched for a positive or negative evolutionary signature of ADAR in the two available *Malacoherpesviridae* genomes, which are prototypical of the two known viral species infecting either oysters (OsHV-1) or abalones (AbHV-1). Although these two viruses belong to the same viral family and we could assume that they originated form a common ancestor, they shared a limited number of homologous genes and their sequence similarity is quite low [16]. Such genomic divergence makes our finding even more interesting, since we ascertained in both genomes a strong under-representation of the TA motif (Fig. 8), which we reported as the preferential ADAR target in viral genomes (Fig. 6). This finding suggests that *Malacoherpesviridae* genomes have evolved to limit their susceptibility to the enzymatic activity of bivalve and gastropod ADAR1s. Intriguingly, half of the viral genes analyzed have maximized the TA reduction, meaning that, for these genes, a further TA reduction would modify their coding sequence, resulting in proteins possibly dysfunctional. Unfortunately, in this work we couldn’t predict if modifications of amino acid residues can influence the viral protein function, due to the very limited knowledge about the functional role of *Malacoherpesiviridae* proteins.

## Conclusions

We reported for the first time an extensive ADAR-mediated editing impacting RNAs expressed by the only two known members of the *Malacoherpesviridae* family (dsDNA viruses). The overall results, including phylogenetic analysis, structural modelling, expression profiles, and inference of the functional consequences of the ADAR activity on viral RNAs, suggest an antiviral function of ADAR1 in two lophotrochozoan species, *Crassostrea gigas* and *Haliotis diversicolor supertexta*. Differently from nematodes and arthropods, which primarily use RNA-interference as antiviral resistance mechanism [87], lophotrochozoans greatly rely on an interferon-like pathway to counteract viruses [33]. The evidence of a biological role of ADAR1, a gene possibly controlled by such pathway, a can be regarded as proof of concept that ADAR1 editing, long-lasting in evolutionary terms, has contributed to evolve viral genomes limiting the ADAR editing to few hotspots. To establish whether *Malacoherpesviridae* have absorbed the originally adverse activity of ADAR1 and have directed the ADAR-editing to beneficially impact their own replication or if ADAR1 still currently exert an antiviral activity, requires further study.

## Methods

### Data retrieval and identification of ADAR sequences

Gene models of genome-sequenced lophotrochozoans were downloaded from the Ensembl Metazoa release 39 (*C. gigas,* GCA_000297895.1; *Lingula anatina,* GCA_001039355.1; *O. bimaculoides,* GCA_001194135.1; *Lottia gigantea,* GCA_000327385.1; *Capitella teleta,* GCA_000328365.1 and *Helobdella robusta, GCA_000326865.1*) or from other public repositories (*C. virginica, Pinctada fucata, Mytilus galloprovincialis, Bathymodiolus platifrons, Modiolus philippinarum, Mizuhopecten yessoensis, Phoronis australis and Notospermus geniculatus* [88–92]). To identify *ADAR-like* genes we searched for the predictive A_deamin domain (PFAM ID: PF02137) using HMMer v.3.1 [93] applying an E-value cut-off of 10^− 5^. Subsequently, *hmmscan* was used to identify conserved Pfam-A v.29 domains [94] on the identified proteins, applying the same E-value as above.

### Alignment, phylogenetic analysis and structural modelling

Protein sequences were aligned with MUSCLE [95], applying default parameters. ModelTest-ng v.0.1.2 [96] identified the WAG+F + G substitution model as the best-fitting model of molecular evolution for this multiple sequence alignment. Accordingly, a Bayesian phylogenetic analysis was performed using MrBayes v.3.2.5 [97]. Two separate Markov Chain Monte Carlo analyses were run in parallel with four chains each for 1,000,000 generations, with a sampling frequency of 1,000 and a burn-in of 25% of the sampled trees. The convergence of parallel runs was estimated by reaching an average standard deviation of split frequency < 0.05 and of a potential scale reduction factor equal to 1. Adequate posterior sampling was evaluated by reaching an effective sample size > 200 for each of the estimated parameters using Tracer v.1.6 [98]. The resulting phylogenetic tree was visualized and edited using FigTree v.1.4.3 (http://tree.bio.ed.ac.uk/software/figtree/), whereas the alignment is available as Additional file 7.

Both the full-length sequence and the catalytic domain of *C. gigas* EKC29721 have been modelled by Swiss Model server (https://swissmodel.expasy.org). Other homology modelling servers have been queried (giving very similar results they haven’t been used for the subsequent analysis). For our purpose, the most robust and reliable results were obtained for the catalytic domain (see Additional file 8 for further details). The model, covering 98% of the submitted sequence (residue:298–661), was built by target-template alignment using ProMod3 and fragments libraries to remodel insertions and deletions, and has been evaluated by the GMQE function (global model quality estimation) and overall QMEAN (qualitative model energy analysis) scoring parameter, resulting in 0.62 and − 2.80 respectively. Geometrical parameters calculated for the resulting model such as the interaction potential between both Cβ (− 1.90) and all atoms (− 2.06) in the structure, the solvation potential (− 0.34) and the torsion angle potential (− 2.40), which contribute to determine the global QMEAN suffer the low resolution of majority of the best templates (5ED1, 5ED2, 5HP2, 5HP3).

### Animal sampling, viral DNA quantification, RNA extraction and cDNA preparation

*C. gigas* specimens were sampled in the lagoon of Goro (North Adriatic Sea, Italy) in May 2016. Total flesh of 15 individual oysters (samples S1-S15) was divided in two subsamples, the first one was immediately homogenized in 1 ml Trizol (Life Technologies, Germany) using a T-10 Ultra-Turrax (IKA, Staufen, Germany) and frozen at − 80 °C until RNA extraction, while the second part was subjected to molecular diagnosis of OsHV-1, according to [99]. The processing of *H. diversicolor supertexta* samples was described in detail elsewhere, including also the analysis of host and viral expression profiles [68, 78]. Briefly, the abalones originated from a Chinese farming area and were sampled in the frame of a large AbHV-1 infection trial. The RNA-seq samples originated from time zero, 24 and 60 hpi points of a 0–72 h challenge with an infective homogenate of AbHV-1-CN2003. Total RNA was extracted according to Trizol manufacturer’s instructions, quantified using the Qubit RNA BR Assay Kit (Life Technologies, Carlsbad, USA) and checked for quality using an Agilent Bioanalyzer 2100 Nano kit (Agilent, Santa Clara, USA). cDNA was prepared by retro-transcription of 1 μg of total RNA, using oligo (dT)12–18 primer (25 ng) and 200 U of SuperScript II Reverse Transcriptase (Life Technologies), diluted 1:5 and used for qRT-PCR analysis.

### Real time quantitative PCR analysis

qRT-PCR reactions were carried out starting from 1 μl of cDNA in 15 μl of final reaction mixture using the DyNAmo HS SYBR Green qPCR kit (Thermo Scientific, Waltham, USA). The housekeeping gene *Elongation factor 1 alpha* (EF1-α) was chosen as reference for *C. gigas*, whereas the *Y-box binding protein 1* was used for *H. diversicolor*, since it was verified as reliable housekeeping gene in a previous study [100]. OsHV-1 ORF104 and its AbHV-1 homolog, ORF68, were used as proxy to determine the overall viral transcription according to their robust expression trend previously reported [76, 78]. Primer pairs (Table 5) were designed using Primer 3 (http://bioinfo.ut.ee/primer3-0.4.0/). Amplification cycles were performed in triplicate using an Applied Biosystems 7900HT Fast Real-Time PCR System with the following cycle: 95 °C for 15 min and 40 cycles of 95 °C for 30 s and 60 °C for 1 min. The relative expression ratio of the selected target gene (RQ) was based on the delta–delta Ct method (2^−ΔΔCt^) [101].

**Table 5:** Primer pairs used for qRT-PCR assays. Organism, gene symbol, forward and reverse primer sequences and amplicon size are reported

Organism	Gene	NCBI ID	Primer F	Primer R
*C. gigas*	El1α	NM_001305313	GCATTTTGGTGCCTCTTCCA	ACCACCCTGGTGAGATCAAG
	CgADAR1v	XM_011448062	TTATATGGCTGCCTGTCT	GCTCGTATTTCCCCATTT
*H. diversicolor*	YB1	JN997407	AAGTTCTAGCAACGAGGGTCA	GGTATTTCTTTGGGTTGTTCTTC
	HdADAR1	MH708893	AAGATGGAGGGAGGTGAAG	AGTGAGTCGAGATATATGGGT
	HdADAR2a	MH708891	GTGAGAGCGGAAGTGAGA	GATGACAGGTACAGAGGG
	HdADAR2b	MH708892	AAGATGGAGGGAGGTGAAG	AGTGAGTCGAGATATATGGGT
	HdADAD	MH708894	GATGAAGATGGTGTTGTG	AGGAAGTGAGTTAAGAGTG
OsHV-1	ORF104	MG561751.2	CAAAGAGCGTGACAAAGGGAA	AAGGAGAGGTTTGAGGATTGG
AbHV-1	ORF68	JX453331	CTACTCTCCTTCTCACCAAC	ATTGCTCATACCTCCCTT

### Library preparation and high-throughput RNA sequencing

A total of 1 μg of total RNA of one *C. gigas* individual sample (S15) was subjected to ribosomal rRNA depletion procedure (Ribo Zero, Illumina, San Diego, USA) and sequenced applying a stranded and paired library layout with an Illumina Hi-Seq2000 machine (2 × 125 bp reads, DNA Sequencing Center, Brigham Young University, USA). *C. gigas* reads are available in the SRA archive (https://www.ncbi.nlm.nih.gov/sra) under accession ID PRJNA484109. *H. diversicolor supertexta* data are available under accession ID PRJNA471241 [68, 78].

### Bioinformatics analysis

If not differently indicated, all the analyses were performed using CLC Genomic Workbench v.10.0 (Qiagen, Hilden, Germany). Additional Illumina reads were obtained to cover all the available oyster and abalone biological samples referring to productive Malacoherpesvirus infections, namely a single *C. gigas* sample referring to environmental infected oyster spat [17] and to an experimental infection trial using susceptible and resistant oyster families [23]. Additional 159 oyster samples were also considered (Additional file 8). OsHV-1-PT and AbHV-1-CN2003 genomes were obtained from the NCBI database [77, 78]. Illumina reads were trimmed for quality, allowing a maximum of two ambiguous bases, minimal quality threshold of Q20, minimal read length of 80 bp and considering only validated pairs using Trimgalore version 0.4.4 (https://www.bioinformatics.babraham.ac.uk/projects/trim_galore/). RNA-seq expression analysis was carried out by mapping the clean reads of each dataset on the *C. gigas* and OsHV-1 annotated genomes, setting length and similarity parameters to 0.8 and 0.9, respectively. Starting from the read counts, Transcripts Per Million (TPM) expression values were computed according to [102]. To estimate the fraction of reads produced from the transcription of *Malacoherpesviridae*, the clean reads of the were mapped to the known *Malacoherpesviridae* genomes (Table 3), and the total number of mapped reads were extracted and tabulated.

To detect genuine SNVs, the clean reads were mapped on *C. gigas* and OsHV-1-PT genomes (oyster samples) or on the AbHV-1-CN2003 genome only (abalone samples). The mapping on the oyster genome was performed with the *large gap read mapping* (LGRM) tool (0.8 and 0.8 for length and similarity fraction, respectively), whereas a *simple mapper* tool was used to map reads on viral genomes (0.5 and 0.8 for length and similarity fraction, respectively). SNV calling was performed on the mapping files and nucleotide changes were called “SNV” if present in at least 1% of the locally aligned reads using the following parameters: minimum required coverage, 20x; minimum required count, 4. Transcriptome *de-novo* assemblies were performed using the CLC *assembler* tool, setting word and bubble size parameters to “automatic” and considering a minimal contig length of 200 bp. The assembled contigs were subjected to ORF prediction by the *transdecoder* tool (Trinity suite [103]), with a minimal protein length of 100 residues.

### Viral genome vulnerability analysis

Under-representation and replacement transition fraction analysis were performed using the n3 module of the *Cytidine Deaminase Representation Reporter* (CDUR) [81]*.* Briefly, this reporter received as input a coding sequence. This input was shuffled 1000 times by switching nucleotides in the third positions of the codons such that the integrity of the underlying amino-acid sequence was not compromised. Unaltered GC content of the input and shuffled genomes was worthy of note, as the GC content itself has been shown to play a role in the under-representation of hotspots [45]. We measured the relevant “belowXX” and “repTrFracXX” statistics (e.g. belowTA and repTrFracTA). The “below” metrics counted the number of hotspots (e.g., TA) in the input and compared this number to the distribution of hotspots observed in the shuffled sequences in order to obtain an empirical *P*-value. The replacement transition fraction, or “repTrFrac,” instead compared the ratio of possible non-synonymous mutations that can occur at the hotspot (e.g., TA) to the observed number of hotspots, obtaining a P-value in a similar way. This fraction was compared to the distribution resulting from the shuffled sequences, to obtain a second empirical P-value.

## Additional files

Additional file 1: Multiple alignment of the A_deamin domain sequences used for the phylogenetic analysis. Species names are abbreviated according to Fig. 1. (ALN 37 kb)

Additional file 2: **Figure S1.** Distribution of ADAD, ADAR1 and ADAR2 proteins in the 14 lophotrochozoan genomes analyzed. The different protein types were classified according to their domain composition. (TIF 1136 kb)

Additional file 3: **Figure S2.** Expression profiles of *C. gigas* ADAR genes computed in 202 RNA-seq samples (listed in Additional file 8). Expression values (as TPM values) of the four CgADARs are reported for each RNA-seq sample. The number of reads mapped to the OsHV-1 genome are reported on the secondary Y-axis. Raw data are included in Additional file 9. (TIF 4182 kb)

Additional file 4: **Figure S3.** SNV hotspots in *Malacoherpesviridae* genomes. A. Along the OsHV-1 genome (207 kb in length) we report the position of the viral ORFs (yellow arrows according to their coding directionality), the coverage graph of the RNA reads mapped according to the ORF directionality (R reads) or on the opposite strand (F reads) as well as the SNV distributions computed separately using the 2 read subsets (R and F reads). B. The circular graph summarizes the distribution of the different SNV types of the R and F SNVs. C. Along the AbHV-1 genome (represented by 5 joined contigs, contig 0–4) we report the position of the viral ORFs (yellow arrows according to their coding directionality) and the SNV distributions in 3 samples (MA49–51). (TIF 5471 kb)

Additional file 5: **Figure S4.** SNV distribution in the OsHV-1-PT genome. (PDF 252 kb)

Additional file 6: **Figure S5.** *C. gigas* SNV profile. The graph reports the frequency of the different SNV types impacting the oyster protein-coding gene models (*C. gigas* CDS, green bars), the S15 *de-novo* assembled coding transcript (S15 CDS, orange bars), UTRs (S15 UTR, grey bars) and ncRNAs (S15 ncRNA, blue bars). (TIF 1583 kb)

Additional file 7: Computational details of the *C. gigas* ADAR1 structural modeling. (PDF 457 kb)

Additional file 8: *C. gigas* RNA-seq samples used for *in-silico* expression analysis. (XLSX 17 kb)

Additional file 9: TPM expression values of oyster and abalone ADARs in the analyzed RNA-seq samples (202 for *C. gigas* and 9 for *H. diversicolor supertexta*). (XLSX 17 kb)

## Data Availability

The datasets generated during the current study are available in the SRA archive, under project accession ID PRJNA484109 (*C. gigas* sample) and PRJNA471241 (*H. diversicolor supertexta* samples). All the analyzed data during this study are included in this published article and in its Additional information files.

## Abbreviations

dsDNA: Double-stranded DNA
HTS: High-throughput sequencing
ISG: Interferon stimulated gene
kDA: kilo Dalton
ncRNA: non-coding RNA
qRT-PCR: Quantitative real-time polymerase chain reaction
SNV: Single nucleotide variation
TPM: Transcript per million
UTR: Untranslated region
