# Supplementary figures and images for: A-to-I editing of Malacoherpesviridae RNAs supports the antiviral role of ADAR1 in mollusks
Source: BMC Evol Biol. 2019 Jul 23;19:149. doi: 10.1186/s12862-019-1472-6 (PMC6651903; doi:10.1186/s12862-019-1472-6)

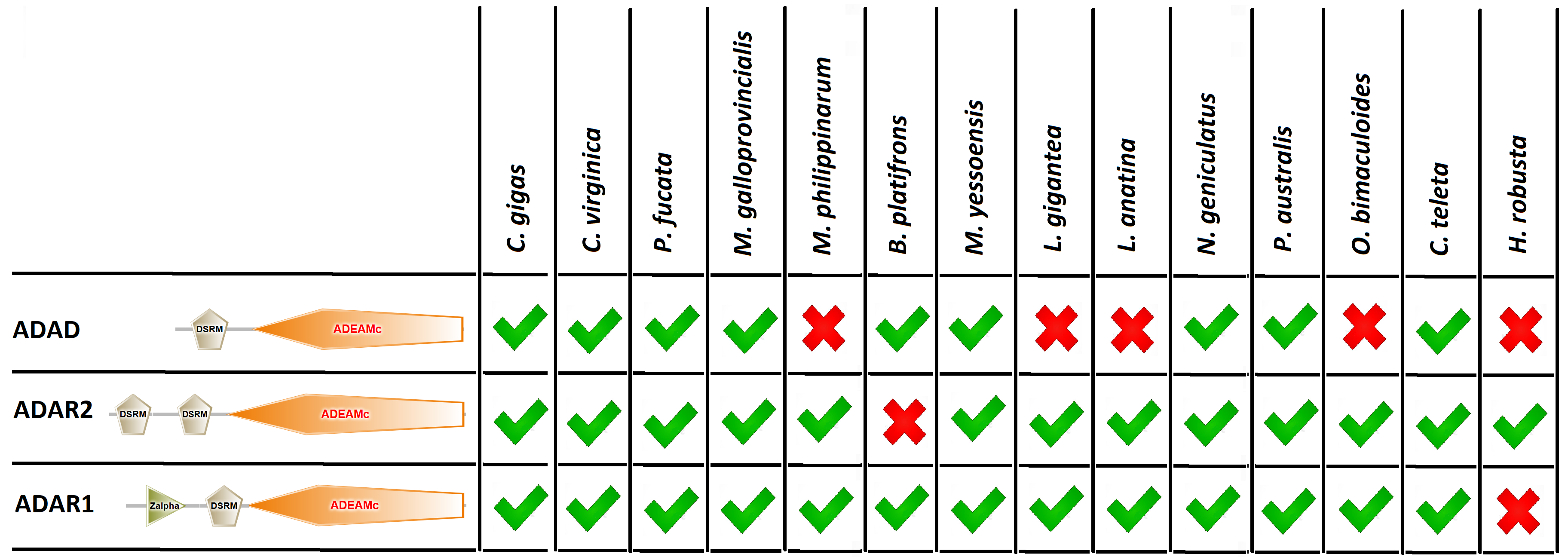

Supplement: Supplementary file 2 — Figure S1. Distribution of ADAD, ADAR1 and ADAR2 proteins in the 14 lophotrochozoan genomes analyzed. The different protein types were classified according to their domain composition. (TIF 1136 kb) [file 12862_2019_1472_MOESM2_ESM.tif]

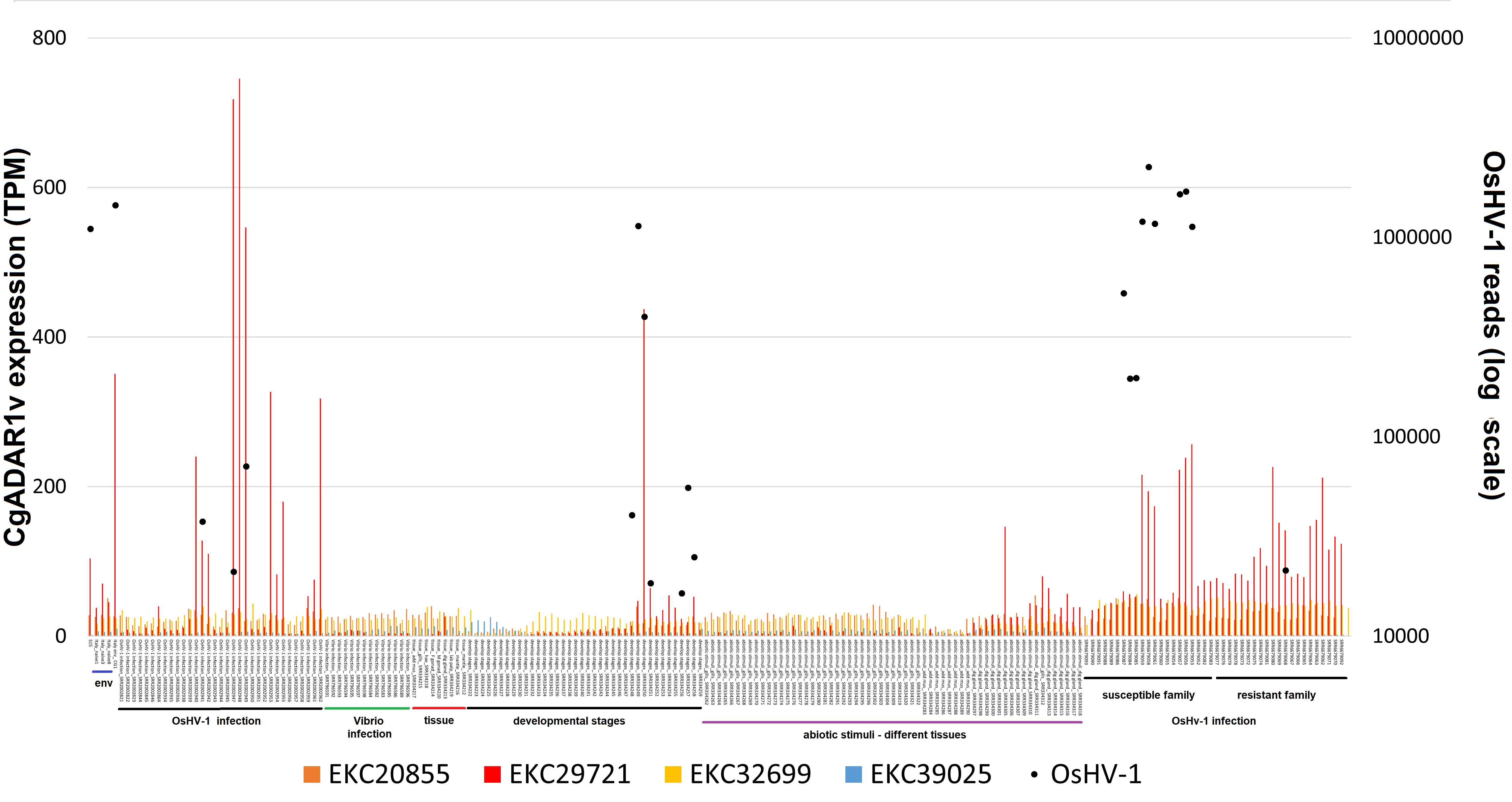

Supplement: Supplementary file 3 — Figure S2. Expression profiles of C. gigas ADAR genes computed in 202 RNA-seq samples (listed in Additional file 8). Expression values (as TPM values) of the four CgADARs are reported for each RNA-seq sample. The number of reads mapped to the OsHV-1 genome are reported on the secondary Y-axis. Raw data are included in Additional file 9. (TIF 4182 kb) [file 12862_2019_1472_MOESM3_ESM.tif]

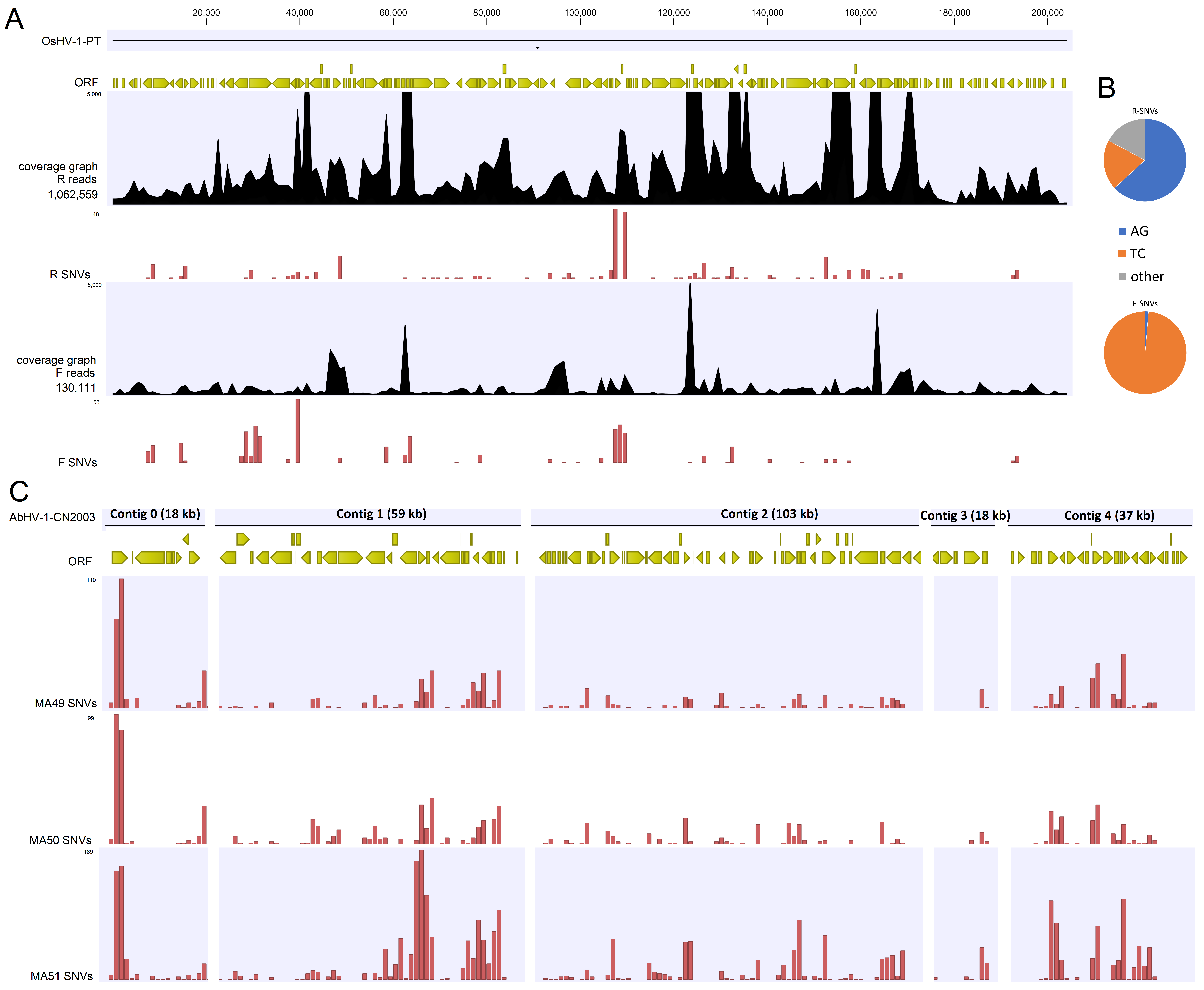

Supplement: Supplementary file 4 — Figure S3. SNV hotspots in Malacoherpesviridae genomes. A. Along the OsHV-1 genome (207 kb in length) we report the position of the viral ORFs (yellow arrows according to their coding directionality), the coverage graph of the RNA reads mapped according to the ORF directionality (R reads) or on the opposite strand (F reads) as well as the SNV distributions computed separately using the 2 read subsets (R and F reads). B. The circular graph summarizes the distribution of the different SNV types of the R and F SNVs. C. Along the AbHV-1 genome (represented by 5 joined contigs, contig 0–4) we report the position of the viral ORFs (yellow arrows according to their coding directionality) and the SNV distributions in 3 samples (MA49–51). (TIF 5471 kb) [file 12862_2019_1472_MOESM4_ESM.tif]

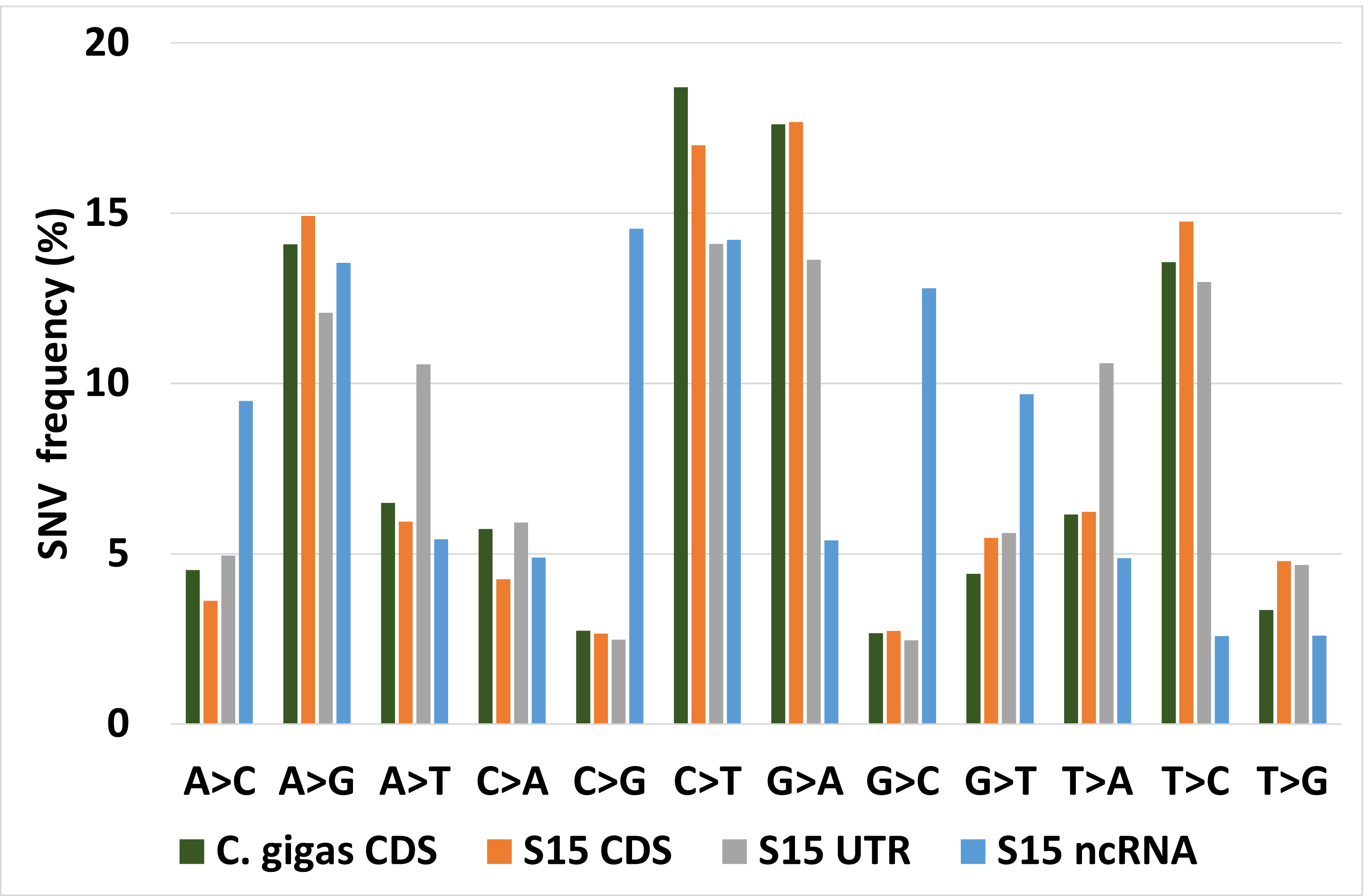

Supplement: Supplementary file 6 — Figure S5. C. gigas SNV profile. The graph reports the frequency of the different SNV types impacting the oyster protein-coding gene models (C. gigas CDS, green bars), the S15 de-novo assembled coding transcript (S15 CDS, orange bars), UTRs (S15 UTR, grey bars) and ncRNAs (S15 ncRNA, blue bars). (TIF 1583 kb) [file 12862_2019_1472_MOESM6_ESM.tif]
