# Supplementary material for: A-to-I editing of Malacoherpesviridae RNAs supports the antiviral role of ADAR1 in mollusks
Source: BMC Evol Biol. 2019 Jul 23;19:149. doi: 10.1186/s12862-019-1472-6 (PMC6651903; doi:10.1186/s12862-019-1472-6)

Additional file 5: Figure S4. SNV distribution in the OsHV-1-PT genome. A. Proof of concept visualization of directional read analysis. Viral RNA reads are mapped on the genome according to the coding direction of the viral ORFs (yellow arrows). The coverage profiles of the reads mapped on a forward ORF (ORFa) are turquoise, whereas the coverage profiles of a reverse ORFs (ORFb and ORFc) are in blue-gray. Blue arrows depicted polyadenylation sites. The SNV calling algorithm revealed either A-to-G SNVs, supported by reads mapping according to coding direction, or T-to-C SNVs, supported by reads mapping on overlapping antisense ORF regions. **B**, **C**, **D**, **E** and **F** panels show example cases of viral RNA reads mapping on the OsHV-1-PT genome. a. viral genome; b. viral ORFs depicted by yellow arrows; c. A-to-G SNVs; d. T-to-C SNVs are reported by red lines; e. polyadenylation signals; f. coverage profile: genomic-forward reads mapped on a given ORF (light-blue), genomic-reverse mapped reads, according to panel A (blue), fraction of the coverage graph related to reads mapped as unpaired (red), fraction (if present) of reads mapping in multiple locations (yellow). **B.** SNV hotspot located between ORF6 and ORF7. The coverage graphs relative to each gene showed a clear overlapping between the two UTRs, a region characterized by numerous A-to-G and T-to-C SNVs. **C.** The longer 5′-UTR of ORF10 overlapped the shorter 5′-UTR of ORF9. Also, we detected the presence of 3′ polyadenylation signals (reported in blue), corresponding to coverage drops-offs consistent with the end of transcription. **D.** Overlapping 3'UTRs (ORF22 and ORF23). The 3′ polyadenylation signals of ORF22 and ORF23 are located near the end of their coding sequences, resulting in no antisense transcript-overlap and, accordingly, no ADAR-compatible SNVs were detected in this region. **E.** ORFs oriented in the same direction, showing different expression levels but no SNVs. **F**. Genomic region of ORF72 characterized by ADAR hyper-editing, distributed on both strands without a clear explanation of the possible origin of such SNVs. Except for the well-defined coverage peak on ORF72, both the read-directional coverage graphs suggest a certain level of transcription beyond the annotated ORFs.

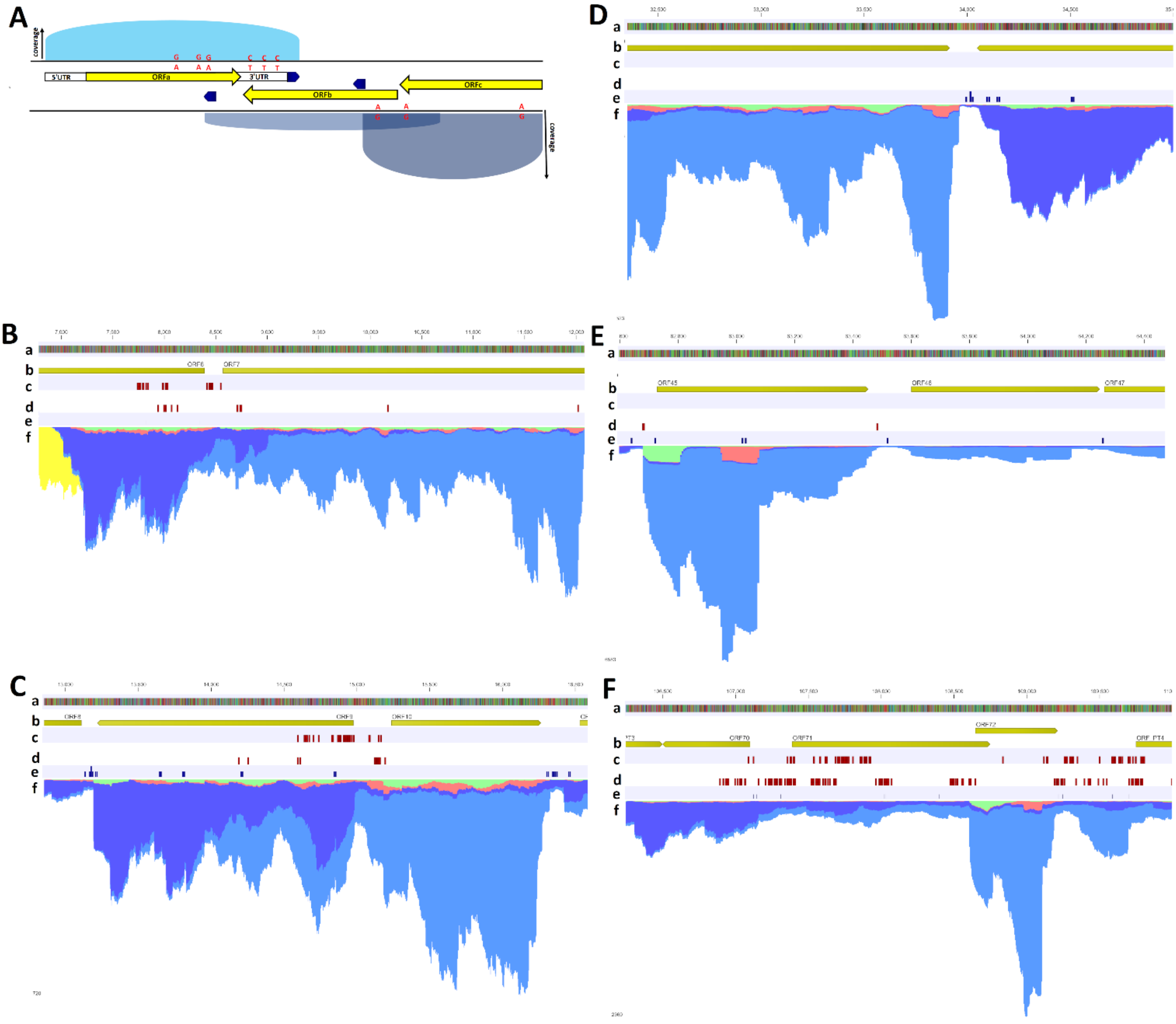

Supplement: Supplementary file 5 — Figure S4. SNV distribution in the OsHV-1-PT genome. (PDF 252 kb) [file 12862_2019_1472_MOESM5_ESM.pdf]
