# Supplementary material for: A-to-I editing of Malacoherpesviridae RNAs supports the antiviral role of ADAR1 in mollusks
Source: BMC Evol Biol. 2019 Jul 23;19:149. doi: 10.1186/s12862-019-1472-6 (PMC6651903; doi:10.1186/s12862-019-1472-6)
Supplement: Supplementary file 7 — Computational details of the C. gigas ADAR1 structural modeling. (PDF 457 kb) [file 12862_2019_1472_MOESM7_ESM.pdf]

Additional File 2. Computational details of the *C. gigas* ADAR1 structural modeling.

List of templates identified by SWISS MODEL SMTL library search

(SMTL version 2018-07-12, PDB release 2018-07-06)

Template	Seq Identity	Oligo-state	Found by	Method	Resolution	Coverage	Description
5ed2.1.A	38.46	monomer	BLAST	X-ray	2.95Å	0.81	Double-stranded RNA-specific editase 1
5ed1.2.A	38.46	monomer	BLAST	X-ray	2.77Å	0.81	Double-stranded RNA-specific editase 1
5ed1.1.A	38.46	monomer	BLAST	X-ray	2.77Å	0.81	Double-stranded RNA-specific editase 1
5hp3.1.A	38.40	monomer	HHblits	X-ray	3.09Å	0.80	Double-stranded RNA-specific editase 1
5hp2.2.A	38.74	monomer	BLAST	X-ray	2.98Å	0.81	Double-stranded RNA-specific editase 1
5hp3.1.A	38.74	monomer	BLAST	X-ray	3.09Å	0.81	Double-stranded RNA-specific editase 1
1zy7.1.A	38.74	monomer	BLAST	X-ray	1.70Å	0.81	RNA-specific adenosine deaminase B1, isoform DRADA2a
1zy7.2.A	38.74	monomer	BLAST	X-ray	1.70Å	0.81	RNA-specific adenosine deaminase B1, isoform DRADA2a
3vyx.1.A	21.88	monomer	HHblits	X-ray	2.29Å	0.14	ATP-dependent RNA helicase A
2l2n.1.A	33.33	monomer	HHblits	NMR	NA	0.13	Hyponastic leave 1
1uil.1.A	20.00	monomer	HHblits	NMR	NA	0.14	Double-stranded RNA-binding motif
2rs7.1.A	20.00	monomer	HHblits	NMR	NA	0.14	ATP-dependent RNA helicase A
3adi.3.A	32.20	monomer	HHblits	X-ray	3.20Å	0.13	F21M12.9 protein
3adi.2.A	32.20	monomer	HHblits	X-ray	3.20Å	0.13	F21M12.9 protein
3adi.1.A	32.20	monomer	HHblits	X-ray	3.20Å	0.13	F21M12.9 protein
3adg.1.A	32.20	monomer	HHblits	X-ray	1.70Å	0.13	F21M12.9 protein
2n3f.1.A	23.33	monomer	HHblits	NMR	NA	0.13	Double-stranded RNA-binding protein 4
2n3f.1.A	28.33	monomer	HHblits	NMR	NA	0.13	Double-stranded RNA-binding protein 4
2n3h.1.A	23.33	monomer	HHblits	NMR	NA	0.13	Double-stranded RNA-binding protein 4

List of templates identified by SWISS MODEL SMTL library search

(SMTL version 2018-07-12, PDB release 2018-07-06)

5aor.2.A	18.75	monomer	HHblits	X-ray	2.08Å	0.14	DOSAGE COMPENSATION REGULATOR
2n3g.1.A	28.33	monomer	HHblits	NMR	NA	0.13	Double-stranded RNA-binding protein 4
1qu6.1.A	31.67	monomer	HHblits	NMR	NA	0.13	PROTEIN KINASE PKR
1x49.1.A	25.00	monomer	HHblits	NMR	NA	0.13	Interferon-induced, double-stranded RNA-activated protein kinase
5mrc.31.A	23.33	monomer	HHblits	EM	NA	0.13	mL44
2ez6.1.C	23.73	monomer	HHblits	X-ray	2.05Å	0.13	Ribonuclease III
1yyo.1.F	23.73	monomer	HHblits	X-ray	2.90Å	0.13	Ribonuclease III

Alignment of *C. gigas* ADAR1 C-terminal domain with the best template structure identified by SWISS-MODEL server and used in the model building procedures

```
Target     HAQSQHLTCDVIETSPRRGPPHDLTFYMAACLGKQKFKSVTGKNLNEAKNRATAEALRHLKKMGKYELKSSQRTTEEMPH
5ed2.1.A   -------------------------------------------------------------------------------H

Target     TIQTWDDRIAVETLTKFRSLVATENEDLSGRKVLAAILLYDNNE-DELTVVSLGTGNRCITGDHLCVEGTVLNDSHAEII
5ed2.1.A   LPQVLADAVSRLVLGKFGDLTDNFSSPHARRKVLAGVVMTTGTDVKDAKVISVSTGTKCINGEYMSDRGLALNDCHAEII

Target     AKRGFKRYMCEEISRAQTRRGSK---ILMQTSSGTLKVMSHLSFHLYISTAPCGDGAVFTRADP--EDTGRNHAPIFRNR
5ed2.1.A   SRRSLLRFLYTQLELYLNNKDDQKRSIFQKSERGGFRLKENVQFHLYISTSPCGDARIFSPHEPILEEPADRHP---NRK

Target     QHGLLRSKVENGEGTIP--TDDIPQTLDGIRRGQRLRTMSCSDKICRWNVLGLQGALLSQFLEPIYLSSITLGMLFNDGH
5ed2.1.A   ARGQLRTKIESGQGTIPVRSNASIQTWDGVLQGERLLTMSCSDKIARWNVVGIQGSLLSIFVEPIYFSSIILGSLYHGDH

Target     MSRAMCCRVDRDDQPLTGLPQEYRAQHPKLGCVTRVTDTRSVDRSSPISVNWNMADNSVEVTDGTRGMTTRNTPSRLCKR
5ed2.1.A   LSRAMYQRISN----IEDLPPLYTLNKPLLSGISN-AEARQPGKAPNFSVNWTVGDSAIEVINATTGKDELGRASRLCKH

Target     SLFLSYSRIEQNIGHR----------TYRETKDMATEYSAAKGVFESVMDENGYGHWVRKPLEVDLFHL
5ed2.1.A   ALYCRWMRVHGKVPSHLLRSKITKPNVYHESKLAAKEYQAAKARLFTAFIKAGLGAWVEKPTEQDQFSL
```

Models summary: modelling details and quality evaluation of the two top models built based on the best templates (see table below)

Template used	Built with	Oligo-state	Ligands	GMQE	QMEAN
5ed2.1.A	ProMod3 Version 1.1.0	monomer	-	0.62	-2.80

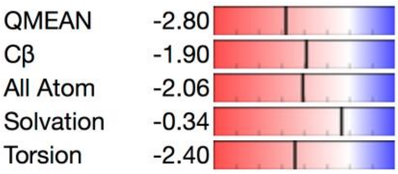

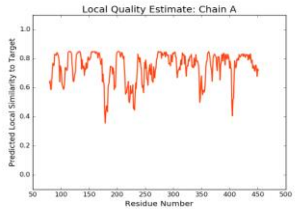

Template	Seq Identity	Oligo-state	Found by	Method	Resolution	Seq Similarity	Range	Coverage	Description
5ed2.1.A	38.46	monomer	BLAST	X-ray	2.95Å	0.40	80 - 451	0.81	Double-stranded RNA-specific editase 1

Ligand	Added to Model	Description
IHP	✕ - Binding site not conserved.	INOSITOL HEXAKISPHOSPHATE
ZN	✕ - Binding site not conserved.	ZINC ION

Template used	Built with	Oligo-state	Ligands	GMQE	QMEAN
5ed1.2.A	ProMod3 Version 1.1.0	monomer	ZINC ION	0.62	-2.97

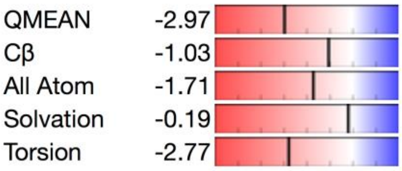

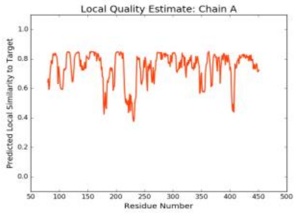

Template	Seq Identity	Oligo-state	Found by	Method	Resolution	Seq Similarity	Range	Coverage	Description
5ed1.2.A	38.46	monomer	BLAST	X-ray	2.77Å	0.40	80 - 451	0.81	Double-stranded RNA-specific editase 1

Ligand	Added to Model	Description
ZN	✓	ZINC ION
IHP	✕ - Binding site not conserved.	INOSITOL HEXAKISPHOSPHATE
